# Helminth parasites of alien freshwater fishes in Patagonia (Argentina)

**DOI:** 10.1016/j.ijppaw.2018.09.008

**Published:** 2018-10-01

**Authors:** Carlos Rauque, Gustavo Viozzi, Verónica Flores, Rocío Vega, Agustina Waicheim, Guillermo Salgado-Maldonado

**Affiliations:** a Laboratorio de Parasitología–INIBIOMA (CONICET–Universidad Nacional del Comahue), Avda. Quintral 1250, 8400, San Carlos de Bariloche, Río Negro, Argentina; b Universidad Nacional Autónoma de México, Instituto de Biología, Apartado Postal 70153, 04510, México D.F, Mexico

**Keywords:** Invasions, Co-introduced, Co-invasive, Exotic fishes, Spillback, Spillover

## Abstract

A survey of the helminth parasites of alien freshwater fishes from Argentinean Patagonia is presented, based on samples taken from 2010 to 2017 and including previous published records. A total of 1129 fishes were collected, belonging to 11 species from 7 families. We surveyed 34 localities in 12 river basins, and found 43 parasite taxa (15 digeneans, 14 monogeneans, 5 cestodes, 5 nematodes, and 4 acanthocephalans), belonging to 22 families. Data are presented as a parasite/host list with information on host species and localities, site of infection, parasite life–history stage, origin, previous records in Patagonia, and accession numbers to vouchers. The most frequently found helminths were monogeneans and digeneans. Our data suggest that invading fish in Patagonia have transmitted fewer parasite species than they have received by spillback. Twenty–three (53%) of the parasites seem to be acquired by the exotic fishes from native hosts, while 15 helminths were co–introduced along with their exotic fish host and continue to parasitize these alien fish but did not invade native hosts; 4 of these species were introduced with carp, 3 with *Cheirodon interruptus*, 3 with *Corydoras paleatus,* 3 with *Cnesterodon decemmaculatus*, 1 with *Oncorhynchus tshawytscha*, and 1 with *Jenynsia multidentata*. The majority of these co–introduced parasites came from the Brazilic ichthyogeographic region (10 species). This is the first review of helminth parasites of alien fishes in Argentina; in total 12 new records of parasites for Argentina, 6 new records of parasites for Patagonia, and 29 new host–parasite records are presented here. This list is far from complete, however, given that some basins in southern Patagonia remain unexplored in terms of parasite detection.

## Highlights

• This is the first review of helminth parasites of alien fishes in Argentina.
• We found 43 parasite taxa in alien fishes, belonging to 22 families.
• We reported 12 new parasite records for Argentina and 6 new records for Patagonia.
• Alien fish in Patagonia have transmitted fewer parasites than they have received by spillback.

## Graphical abstract

**Figure:**
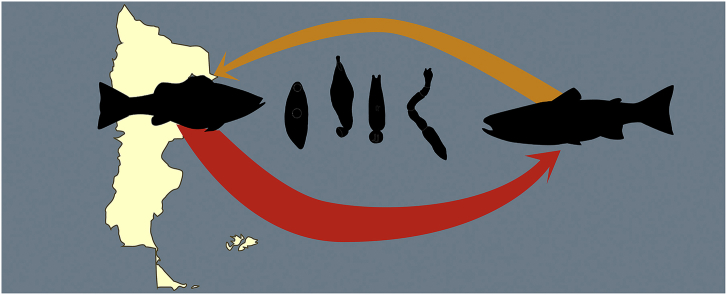

## 1 Introduction

The introduction of species represents a major cause of biodiversity loss, and alteration and homogenization of freshwater ecosystems (Rahel, 2002). The influence of processes such as competition and predation of the new invaders on native fauna have been known for a long time (Elton, 1958). Along with invasion or the introduction of species into an area, there is often a parallel introduction (a hidden invasion) of parasites (Salgado–Maldonado and Pineda–López, 2003). Parasites account for nearly a quarter of IUCN's list of invasive species (Dunn and Perkins, 2012). The introduction of parasites can lead to novel host–parasite relationships changing the structure of pre–existent communities (Poulin, 2017). An alien parasite can disturb native host populations, depending on its ability to infect them, even when they are not phylogenetically close to the original hosts (Lebarbenchon et al., 2009; Telfer and Bown, 2012). The magnitude of the threat posed to native species will be related mostly to parasite virulence and pathogenicity (Lymbery et al., 2014). Co–introduced parasites are those which have been transported with an alien host to a new locality outside their natural range, and co–invading parasites are those which have been co–introduced and then spread to new, native hosts (Lymbery et al., 2014) in a process called spillover (Kelly et al., 2009). On the other hand, native parasites may interact with exotic hosts, leading to a process known as spillback (Kelly et al., 2009; Poulin, 2017). Lymbery et al. (2014) found that more than 50% of invasion studies were on freshwater fish, and 49% of co–introduced were helminths. Seventy–eight percent of the co–introduced parasites were found in native fish and therefore can be classified as co–invaders. Ecologists and conservation managers have become increasingly aware of the threat posed by co–introduction of parasites along with alien hosts (Kelly et al., 2009).

**Fig. 1:**
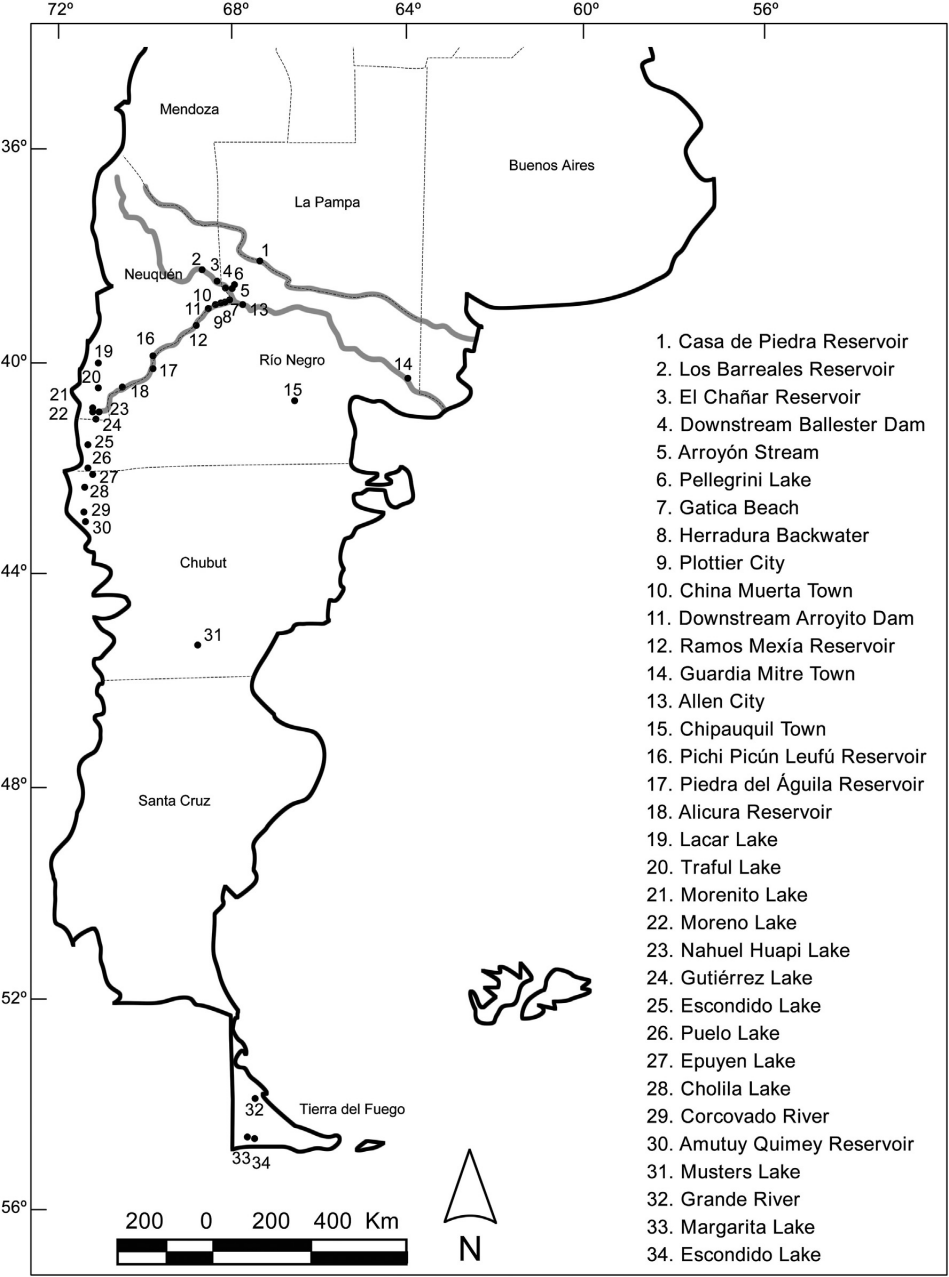
Map of Argentinean Patagonia; the sampling localities of present study are shown.

Patagonia is a South American territory extending south in Argentina from 37°55′S and covering about 1,000,000 km^2^ (Fig. 1). Freshwater environments in this region can be classified into two groups: those close to the Andes Mountains are oligotrophic or ultraoligotrophic, belong to the Atlantic or Pacific watershed, and have cold, well–oxygenated water with low conductivity (Modenutti et al., 1998). These freshwater environments are surrounded by Sub Antarctic forests, which are the southernmost forests in the world. The remaining aquatic environments on the Patagonian plateau, also known as the Patagonia Desert, are mainly rivers or reservoirs that belong to the Atlantic watershed, have warmer, less–oxygenated water and higher conductivity. The Colorado River is the northern limit of the Austral subregion and the Negro River represents the southern limit of the Brazilian subregion, so there is an overlapping of Brazilian and Austral fish–fauna in the area between the two rivers (Almirón et al., 1997; Aigo et al., 2008). These rivers are the watersheds most likely to be influenced by introductions from northern ichthyogeographical provinces. The expected global changes for northern Patagonia could affect the provision of key ecosystem services at regional, national and global levels. For example, on a regional level the provision of aesthetic–recreational services by the Andean–Patagonian landscapes is key to sustaining the tourism industry. Climatic changes affecting the water regime, temperature and rainfall, along with anthropogenic alterations, favor biological invasion processes, with serious consequences for ecosystems (Sala et al., 2000).

The fish fauna of Argentinean Patagonia has a low diversity of species, and almost the half of the species are introduced (intentionally and non–intentionally). Some of these alien species are even currently numerically dominant, such as the onesided livebearer *Jenynsia multidentata* (Jenyns) in the Colorado and Negro river basins on the Patagonian plateau, and the rainbow trout, *Oncorhynchus mykiss* (Walbaum), in various lakes and rivers of the Andes Mountains (Alvear et al., 2007; Pascual et al., 2007; Aigo et al., 2008; Cussac et al., 2016). Salmonids were intentionally and extensively introduced into Patagonia as embryos throughout the entire XX century (Macchi et al., 1999; Pascual et al., 2001; Macchi and Vigliano, 2014); in Northern Patagonia at the present time there are self–sustaining populations of *O. mykiss*, the Atlantic salmon *Salmo salar* (Linnaeus), the brown trout *Salmo trutta* (Linnaeus), and brook trout *Salvelinus fontinalis* (Mitchill). Additionally, the Chinook salmon *Oncorhynchus tshawytscha* (Walbaum) has invaded areas in southern Patagonia as a result of escapes from Chilean aquaculture (Wegrzyn and Ortubay, 2006; Di Prinzio and Pascual, 2008; Cussac et al., 2016), and the lake trout *Salvelinus namaycush* (Walbaum) has become established in some lakes of Santa Cruz Province (Wegrzyn and Ortubay, 2006). The common carp *Cyprinus carpio* (Linnaeus) was introduced from Europe into Central Argentina (Rosso, 2006). In the 1980s this species invaded Colorado River, the northernmost river basin of Patagonia, probably due to an extraordinary flood of Salado River (López Cazorla and Sidorkewicj, 2002). At the beginning of the 2000s, common carp were recorded in Negro River, constituting its southernmost distribution (Alvear et al., 2007). It is thought that this species could have been introduced into the river by inhabitants of the region in the year 2002, in the locality of Luis Beltrán (39°19′S, 65°46′W), and this species rapidly colonized upstream environments of the basin (Pérez and López Cazorla, 2008). The Argentinean silverside *Odontesthes bonariensis* (Valenciennes) was transplanted into Patagonia as embryos for sportfishing in the middle of last century (Baigún and Ferriz, 2003; Rosso, 2006; Tombari and Volpedo, 2008; Amalfi, 2009). Moreover, another 9 species from the Brazilic ichthyogeographical sub–region accidentally invaded Northern Patagonia when they were used as bait and sold for aquarism (Pérez and López Cazorla, 2008): the mojarritas *Astyanax eigenmanniorum* (Cope) and *Astyanax pampa* Casciotta, Almirón and Azpelicueta, the tetra *Cheirodon interruptus* (Jenyns), the dientudo *Oligosarcus jenynsii* (Günther), the peppered corydoras *Corydoras paleatus* (Jenyns), the livebearers *Jenynsia alternimaculata* (Fowler) and *J. multidentata,* the ten–spotted livebearer *Cnesterodon decemmaculatus* (Jenyns), and the cabeza amarga *Crenicichla lacustris* (Castelnau) (Almirón et al., 1997; Liotta, 2006; Alvear et al., 2007; Pascual et al., 2007; Crichigno et al., 2016). No accurate information regarding dates and number of introductions are available for these species.

The parasite fauna of only four native freshwater fish species have been comprehensively studied in Patagonia, the glaxiids *Aplochiton zebra* Jenyns (Fernández et al., 2012), and *Galaxias maculatus* (Jenyns) (Viozzi et al., 2009; Fernández et al., 2010, 2015a, 2015b), the atherinopsid *Odontesthes hatcheri* (Flores et al., 2016), and the percichthyid *Percichthys trucha* (Valenciennes) (Rauque et al., 2003; Viozzi and Brugni, 2003; Brugni and Viozzi, 2006; Vega et al., 2011). Moreover, notwithstanding the importance of parasite co–introduction into Patagonia freshwater ecosystems, very few studies have been conducted concerning this subject. The first helminth recorded as an alien parasite in Patagonia was the zoonotic cestode *Dibothriocephalus latus* (=*Diphyllobothrium latum*), which was introduced by European immigrants and established in introduced salmonids (Szidat and Soria, 1952; Semenas, 2006). Thereafter, very few parasitological studies were developed on alien fishes, and almost all of these correspond to salmonids and represent isolated reports of some species found in these fishes (Ortubay et al., 1994; Trejo, 1994; Úbeda et al., 1994; Revenga et al., 1995; Semenas, 1998, 2006; Shimazu et al., 2000; Rauque et al., 2003, 2006). Few records concerning scattered localities have been published on the helminth fauna of species of Brazilic origin in Patagonia, mainly in *J. multidentata*, and *O. bonariensis* (Ortubay et al., 1994). Recently, Waicheim et al. (2014) reported helminths of *C. carpio* in one locality of Neuquén River.

In order to control further loss of biodiversity and maintain productive and sustainable ecosystems, it is crucial to understand the ecological mechanisms underlying species invasions, so as to prevent potential harmful effects on native communities. However, important baseline data are largely lacking (Lindegren et al., 2012). With this in mind, the aim of the present work is to produce a checklist of the helminth parasites of alien freshwater fishes which have been introduced into Argentinean Patagonia, by recording data from our own research and compiling information from published records, in order to explore the questions of which parasites have been introduced with these alien fishes, and what the status of these introductions may currently be.

## 2 Materials and methods

Between 2010 and 2017, fishes were collected from 34 localities in 12 river basins in Patagonia (Argentina) (Table 1, Fig. 1). Fish were caught using gill nets, hand nets and seine nets, transported live to the laboratory, measured, and then necropsied to examine fins, skin, eyes, brain, gills, heart, body cavity, liver, gall bladder, stomach, intestine, gonads, and kidney, with the aid of a dissecting microscope. The parasites collected were morphologically studied and identified. Voucher specimens of the majority of the parasite species found as adults in alien fishes were deposited in the Museo Argentino de Ciencias Naturales Bernardino Rivadavia (MACN–Pa), Buenos Aires, Argentina (see Supplementary data).

**Table 1:** Sampling localities, alien fish host species, and number of hosts (between parentheses) examined from 2010 to 2017 for the present survey in Patagonia.

River basin	Locality	Coordinates	Fish species (sample size)
Colorado River	Casa de Piedra Reservoir	38°10′S 67°09′W	*Cyprinus carpio* (3), *Jenynsia multidentata* (44), *Oncorhynchus mykiss* (2), *Odontesthes bonariensis* (5)

Neuquén River	Los Barreales Reservoir	38°32′S 68°49′W	*Oncorhynchus mykiss* (5)
	El Chañar Reservoir	38°34.5′S 68°24′W	*Oncorhynchus mykiss* (2)
	Pellegrini Lake	38°41′S 68°00′W	*Odontesthes bonariensis* (3)
	Arroyón Stream	38°43.66′S 68°02.4′W	*Cheirodon interruptus* (37), *Cnesterodon decemmaculatus* (12), *Corydoras paleatus* (13), *Jenynsia multidentata* (94)
	downstream Ballester Dam	38°43.9′S 68°10′W	*Cyprinus carpio* (55), *Cnesterodon decemmaculatus* (14), *Corydoras paleatus* (3), *Jenynsia multidentata* (22), *Oncorhynchus mykiss* (3)

Limay River	Herradura Backwater	38°57.44′S 68°10.5′W	*Cyprinus carpio* (9)
	Gatica Beach	38°58′S 68°03′W	*Jenynsia multidentata* (2), *Odontesthes bonariensis* (1)
	Plottier City	38°58′S 68°12.7′W	*Jenynsia multidentata* (5)
	China Muerta Town	38°59.5′S 68°19′W	*Cyprinus carpio* (2)
	donwstream Arroyito Dam	39°04.5′S 68°33′W	*Cyprinus carpio* (22), *Jenynsia multidentata* (3), *Oncorhynchus mykiss* (21)
	Ramos Mexía Reservoir	39°26′S 68°56′W	*Oncorhynchus mykiss* (9)
	Pichi Picún Leufú Reservoir	40°01′S 70°00′W	*Salmo trutta* (2)
	Piedra del Águila Reservoir	40°19′S 70°03′W	*Oncorhynchus mykiss* (5), *Salmo trutta* (13)
	Alicura Reservoir	40°35′S 70°50′W	*Oncorhynchus mykiss* (11), *Salmo salar* (6), *Salmo trutta* (9)
	Traful Lake	40°38′S 71°24′W	*Oncorhynchus mykiss* (3)
	Morenito Lake	41°03′S 71°31′W	*Oncorhynchus mykiss* (1)
	Nahuel Huapi Lake	41°05′S 71°20′W	*Oncorhynchus mykiss* (131), *Salmo trutta* (98)
	Moreno Lake	41°05′S 71°30′W	*Oncorhynchus mykiss* (125), *Salmo trutta* (3), *Salvelinus fontinalis* (68)
	Gutiérrez Lake	41°12′S 71°24.5′W	*Oncorhynchus mykiss* (13), *Salmo trutta* (2), *Salvelinus fontinalis* (4)
	Escondido Lake	41°42′S 71°38′W	*Oncorhynchus mykiss* (8)

Negro River	Allen City	39°02′S 67°50′W	*Cyprinus carpio* (52), *Jenynsia multidentata* (10), *Oncorhynchus mykiss* (1)
	Guardia Mitre Town	40°26.5′S 63°41′W	*Cyprinus carpio* (61), *Cheirodon interruptus* (5), *Jenynsia multidentata* (4)

Hua Hum River	Lacar Lake	40°09′S 71°25′W	*Oncorhynchus mykiss* (1)

Valcheta Stream	Chipauquil Town	40°53.6′S 66°32′W	*Cheirodon interruptus* (15), *Cnesterodon decemmaculatus* (15)

Corcovado River	Corcovado River	42°55′S 71°43.1′W	*Oncorhynchus mykiss* (2), *Oncorhynchus tshawytscha* (1)

Puelo River	Puelo Lake	42°06′S 71°37′W	*Oncorhynchus mykiss* (5)
	Epuyén Lake	42°12′S 71°30′W	*Oncorhynchus mykiss* (17), *Salmo trutta* (8)

Futaleufú River	Cholila Lake	42°27.5′S 71°40′W	*Oncorhynchus mykiss* (7), *Salvelinus fontinalis* (8)
	Amutuy Quimey Reservoir	43°04′S 71°41′W	*Oncorhynchus mykiss* (1)

Senguer river	Muster Lake	45°33′S 69°15′W	*Oncorhynchus mykiss* (14)

Grande River	Grande River	53°49′S 67°52.4′W	*Oncorhynchus mykiss* (1)

Azopardo river	Margarita Lake	54°38′S 67°59.4′W	*Oncorhynchus mykiss* (7)
	Escondido Lake	54°39′S 67°48′W	*Oncorhynchus mykiss* (1)

Additionally, records of helminths reported in alien fishes in Patagonia were compiled from scientific literature. Records from theses and scientific meetings, congress summaries and technical reports were excluded as they do not constitute formal publications. The fish species *Percichthys colhuapiensis* MacDonagh and *Percichthys laevis* (Jenyns) are considered morphotypes of *P. trucha* (sensu Ruzzante et al., 2011). Records of parasites determined at a taxonomic level higher than genus were excluded. Parasite taxa are identified as: *first record in Patagonia; # first record in Argentina; † new host record. The checklist includes data of helminth taxa, parasite life–history stage, parasite origin, and host. Data of localities, sites of infection, overall values (pooled localities) of infection for co–introduced or introduced parasites, accession numbers of deposited specimens, other records in Patagonia, and comments about the parasite are given in Supplementary data.

The status of the parasites’ origins were considered as follows: i) parasites found as adults in alien fishes but which have not previously recorded in native fishes are considered **co–introduced**, ii) parasites found as larval stages in alien fishes, whose adults are found in fish–eating birds, and which have been previously recorded in native fishes are classified as **native**, iii) helminth larvae that parasitize fish–eating birds, except for *Goezia* sp. that parasitize fishes as adults, which have not been previously recorded in native fishes are considered **native?** if the alien fish have been introduced as embryonated eggs (salmonids and *O. bonariensis*) because they could not introduce helminths, iv) helminth larvae that parasitize fish–eating birds as adults, but which have not been recorded in native fishes are considered **unknown** if they parasitize fishes which have been introduced as juveniles or adults (e.g. Brazilic fishes), v) helminths introduced by a route other than fishes were classified as **introduced** (the case of species of *Dibotrhiocephalus*).

The checklist follows the classification of Boeger and Kritsky (1993a, b), Thatcher (2006), and Cohen et al. (2013) for monogeneans, Gibson et al. (2002), Jones et al. (2005), and Bray et al. (2008) for digeneans, Khalil et al. (1994) and Alves et al. (2017) for cestodes, Moravec (1998), Anderson et al. (2009), and Gibbons (2010) for nematodes, Amin (2013) for Acanthocephalans, and Froese and Pauly (2017) for fishes. River basins, followed by localities, are presented in latitudinal disposition between brackets, and in cases where localities belong to the same river basin, from upper to lower waters (see Supplementary data).

## 3 Results

In our survey a total of 1129 fishes belonging to 11 species from 7 families were collected; Salmonidae: 396 *Oncorhynchus mykiss*, 1 *Oncorhynchus tshawytscha*, 6 *Salmo salar*, 135 *Salmo trutta*, 80 *Salvelinus fontinalis*; Characidae: 57 *Cheirodon interruptus*; Callichthydae: 16 *Corydoras paleatus*; Cyprinidae: 204 *Cyprinus carpio*; Anablepidae: 184 *Jenynsia multidentata*; Poeciliidae: 41 *Cnesterodon decemmaculatus*; and Atherinopsidae: 9 *Odontesthes bonariensis* (Table 1). All the fish species analyzed were parasitized. We found 43 parasite taxa (15 digeneans, 14 monogeneans, 5 cestodes, 5 nematodes, and 4 acanthocephalans), belonging to 22 families (Table 2). Including previously published records, the complete data set increased to 47 taxa (16 digeneans, 14 monogeneans, 7 nematodes, 6 cestodes, and 4 acanthocephalans). The most frequently found helminths were monogeneans and digeneans, and the most represented families were Dactylogyridae and Gyrodactylidae (Monogenea) with 7 species each, and Diplostomidae (Digenea) with 5 species.

**Table 2:** Helminths from alien fishes examined from 2010 to 2017 for the present survey in Patagonia: parasite stage, origin, and host are given.* First record in Patagonia,# First record in Argentina,† New Host record.

Parasite	Stage	Origin	Host
**Monogenoidea**			
*Characithecium* cf. *costaricensis#*	gravid adults	co–introduced	*Cheirodon interruptus*
*Dactylogyrus anchoratus*#	gravid adults	co–introduced	*Cyprinus carpio*
*Dactylogyrus extensus*	gravid adults	co–introduced	*Cyprinus carpio*
*Diapharocleidus* sp.#	gravid adults	co–introduced	*Cheirodon interruptus*
*Duplaccesorius andinus*	immature adults	native	*Oncorhynchus mykiss*†
*Gyrodactylus anisopharynx#*	gravid adults	co–introduced	*Corydoras paleatus*
Gyrodactylus superbus*	gravid adults	co–introduced	*Corydoras paleatus*
*Gyrodactylus* sp. 1#	gravid adults	co–introduced	*Jenynsia multidentata*
*Gyrodactylus* sp. 2#	gravid adults	co–introduced	*Cheirodon interruptus*
*Gyrodactylus* sp. 3#	gravid adults	co–introduced	*Cnesterodon decemmaculatus*
*Gyrodactylus* sp. 4#	gravid adults	co–introduced	*Cnesterodon decemmaculatus*
*Gyrodactylus* sp. 5#	gravid adults	co–introduced	*Cnesterodon decemmaculatus*
Philocorydoras platensis*	gravid adults	co–introduced	*Corydoras paleatus*
*Pseudacolpenteron* sp.	gravid adults	co–introduced	*Cyprinus carpio*
**Digenea**			
*Acanthostomoides apophalliformis*	immature adults	native	*Oncorhynchus mykiss*
			*Salmo trutta*
			*Salvelinus fontinalis*
	metacercaria		*Jenynsia multidentata*†
*Allocreadium patagonicum*	immature adults	native	*Oncorhynchus mykiss*†
*Ascocotyle* cf. angrense*	metacercaria	native?	*Odontesthes bonariensis*†
*Ascocotyle* cf. diminuta*	metacercaria	unknown	*Jenynsia multidentata*
*Ascocotyle* cf. tertia*	metacercaria	unknown	*Odontesthes bonariensis*†
			*Jenynsia multidentata*
*Derogenes* sp.	gravid adults	native	*Oncorhynchus mykiss*†
			*Salmo salar*†
			*Salmo trutta*†
			*Salvelinus fontinalis*†
*Deropegus patagonicus*	gravid adults	native	*Jenynsia multidentata*†
*Diplostomum* sp.	metacercaria	native	*Oncorhynchus mykiss*
			*Salvelinus fontinalis*
*Posthodiplostomum* sp.	metacercaria	native	*Jenynsia multidentata*
*Pygidiopsis* sp.	metacercaria	native	*Jenynsia multidentata*
			*Odontesthes bonariensis*†
*Steganoderma macrophallus*	gravid adults	native	*Odontesthes bonariensis*†
*Steganoderma szidati*	gravid adults	native	*Oncorhynchus mykiss*†
*Stephanoprora uruguayense*	metacercaria	native	*Cheirodon interruptus*†
			*Cnesterodon decemmaculatus*†
			*Jenynsia multidentata*†
*Tylodelphys* sp. 1*	metacercaria	native?	*Odontesthes bonariensis*
*Tylodelphys* sp. 2	metacercaria	native	*Oncorhynchus mykiss*
			*Salmo trutta*†
			*Salvelinus fontinalis*†
**Cestoda**			
*Ailinella mirabilis*	immature adults	native	*Oncorhynchus mykiss*
			*Salvelinus fontinalis*
*Cangatiella macdonaghi*	gravid adults	native	*Odontesthes bonariensis*
*Dibothriocephalus* sp.	plerocercoid	introduced	*Oncorhynchus mykiss*
			*Salmo salar*
			*Salmo trutta*
			*Salvelinus fontinalis*
*Hepatoxylon* sp.#	plerocercoid	co–introduced	*Oncorhynchus tshawytscha*
*Schizocotyle acheilognathi*	gravid adults	co–introduced	*Cyprinus carpio*
**Nematoda**			
*Camallanus corderoi*	gravid adults in *O. bonariensis*, immature in the other fish species	native	*Oncorhynchus mykiss*
			*Salmo salar*†
			*Salmo trutta*†
			*Salvelinus fontinalis*
			*Jenynsia multidentata*†
			*Odontesthes bonariensis*†
*Contracaecum* sp.	larva	native	*Oncorhynchus mykiss*
			*Salmo salar*†
			*Salmo trutta*
			*Salvelinus fontinalis*†
			*Cyprinus carpio*
			*Cheirodon interruptus*†
			*Cnesterodon decemmaculatus*†
			*Jenynsia multidentata*†
			*Odontesthes bonariensis*
*Hedruris suttonae*	gravid adults	native	*Oncorhynchus mykiss*
			*Salmo trutta*
			*Salvelinus fontinalis*†
*Hysterothylacium patagonense*	immature adults	native	*Oncorhynchus mykiss*
			*Salmo trutta*
			*Salvelinus fontinalis*
*Pseudodelphis limnicola*	immature adults	native	*Oncorhynchus mykiss*†
**Acanthocephala**			
*Acanthocephalus tumescens*	gravid adults	native	*Oncorhynchus mykiss*
			*Salmo trutta*
			*Salvelinus fontinalis*
*Polymorphus* sp. 1#	cystacanth	unknown	*Jenynsia multidentata*
*Polymorphus* sp. 2#	cystacanth	unknown	*Oncorhynchus mykiss*
			*Cyprinus carpio*
			*Cheirodon interruptus*
			*Corydoras paleatus*
*Pomphorhynchus patagonicus*	immature adults	native	*Oncorhynchus mykiss*
			*Salmo trutta*
			*Salvelinus fontinalis*
			*Cyprinus carpio*

More than 53% (23/43) of the parasites seem to be acquired by alien fishes from native hosts, and 15 were co–introduced along with alien fishes: 4 with carp, 3 with *C. interruptus*, 3 with *C. paleatus,* 3 with *C. decemmaculatus*, 1 with *O. tshawytscha*, and 1 with *J. multidentata*. Four species were of unknown origin, and 1 taxon (*Dibothriocephalus* sp.) had been introduced by European immigrants. The majority of co–introduced parasites came originally from the Brazilic ichthyogeographic subregion (10 species), and the remaining helminths came from the Northern Hemisphere, with the exception of 1 taxon (*Hepatoxilon* sp.), which arrived from the Pacific Ocean. The larval nematode *Contracaecum* sp. was the parasite with the widest range of host species (9), followed by the adult nematode *Camallanus corderoi* Torres, Teuber and Miranda, 1990, which occurred in 6 alien fish species. We report 29 new host–parasite records, 12 new parasite records for Argentina, and 6 new parasite records for Patagonia (Table 2). Most of the parasites (29) were found as adults, while 15 were larvae. *Acanthosthomoides apophalliformis* Szidat, 1964 was found in the form of metacercariae in *J. multidentata* and as adults in salmonids.

Almost all the alien fishes examined had acquired native Patagonian parasites (Table 3). The salmonid *O. mykiss* was the fish with the highest parasite species richness, with 17 taxa, of which 88.2% were native. Among the Brazilic fish species, *J. multidentata* showed the highest parasite richness, with 11 taxa, of which 63.6% were native. *Cyprinus carpio* had the lowest value for species richness, and 28.6% of these parasites were native. The only fish species that seemed not to have acquired native parasites were the salmonid O. *tshawytscha* and the callichthyid *C. paleatus* (Table 3).

**Table 3:** Species richness and percentage of native helminths currently recorded in alien fishes in surveys between 2010 and 2017 in Patagonia.

Alien fish	Species richness	number of native helminths
*Oncorhynchus mykiss*	17	15 (88.2%)
*Oncorhynchus tshawytscha*	1	0
*Salmo salar*	4	2 (50%)
*Salmo trutta*	10	8 (80%)
*Salvelinus fontinalis*	12	10 (83.3%)
*Cyprinus carpio*	7	1 (14.3%)
*Odontesthes bonariensis*	8	4 (50%)
*Cheirodon interruptus*	6	1 (16.7%)
*Cnesterodon decemmaculatus*	5	1 (20%)
*Corydoras paleatus*	4	0
*Jenynsia multidentata*	11	5 (45.5%)

### 3.1 Parasite–host list

See Table 2 and Supplementary data.

### 3.2 Host–parasite list

The list of parasites by host is given in Table 4.

**Table 4:** Host–parasite relationships between 12 species of exotic fishes introduced into Patagonia. **1**: Waicheim et al. (2014), **2**: Shimazu et al. (2000), **3**: Ostrowski de Núñez et al. (2017), **4**: Ortubay et al. (1989), **5**: Ortubay et al. (1994), **6**: Flores et al. (2016), **7**: Semenas (1998), **8**: Szidat, Nani (1951), **9**: Ostrowski de Núñez et al. (1999), **10**: Rauque et al. (2003), **11**: Szidat, Soria (1952), **12**: Revenga et al. (1995), **13**: Semenas (2006), **14**: Szidat, Soria (1957), **15**: Szidat (1964), **16**: Revenga, Semenas (1991), **17**: Revenga (1993), **18**: Bacigalupo, D'Alessandro Bacigalupo (1952), **19**: Moravec et al. (1998), **20**: Rauque et al. (2006), **21**: Ortubay et al. (1991), **22**: Semenas et al. (1992), **23**: Trejo (1992), **24**: Úbeda et al. (1994).

	*Oncorhynchus mykiss*	*Oncorhynchus tshawytscha*	*Salmo salar*	*Salmo trutta*	*Salvelinus fontinalis*	*Salvelinus namaycush*	*Cheirodon interruptus*	*Corydoras paleatus*	*Cyprinus carpio*	*Jenynsia multidentata*	*Cnesterodon decemmaculatus*	*Odontesthes bonariensis*
*Dactylogyrus anchoratus*									+			
*Dactylogyrus extensus*									+,1			
*Duplaccessorius andinus*	+											
*Philocorydoras platensis*								+				
*Pseudacolpenteron* sp.									+,1			
*Diapharocleidus* sp.							+					
*Characitecium* cf. *costaricensis*							+					
*Gyrodactylus anysopharynx*								+				
*Gyrodactylus superbus*								+				
*Gyrodactylus* sp. 1										+		
*Gyrodactylus* sp. 2							+					
*Gyrodactylus* sp. 3											+	
*Gyrodactylus* sp. 4											+	
*Gyrodactylus* sp. 5											+	
*Allocreadium patagonicum*	+											
*Deropegus patagonicus*	2			2,3	2,3					+		
*Derogenes* sp.	+		+	+	+							
*Austrodiplostomum mordax*	4,5									5		6
*Diplostomum* sp.	+,5,7		5		+,5	5						
*Posthodiplostomum* sp.										+		
*Tylodelphys* sp. 1												+
*Tylodelphys* sp. 2	+,4,5			+	+					8,5		5
*Stephanoprora uruguayense*							+			+	+	
*Ascocotyle* cf. *angrense*												+
*Ascocotyle* cf. *diminuta*										+		
*Ascocotyle* cf. *tertia*										+		+
*Pygydiposis* sp.										+		+
*Acanthostomoides apophalliformis*	+,5,9,10			+,2	+,10					+		
*Steganoderma macrophallus*												+
*Steganoderma szidati*	+											
*Schizocotyle acheilognathi*									+,1			
*Dibothriocephalus* sp.	+,11,12,13	5,13	+,11,13	+,5,13	+,11,12,13							
*Dibothriocephalus dendriticus*	5,14,15,16,17		5,14,15	16	5,14,16,17							
*Dibothriocephalus latus*	11,14,15,16,17,18		14	16	14,16,17							
*Hepatoxylon* sp.		+										
*Cangatiella magdonaghi*												+
*Ailinella mirabilis*	+,10				+,10							
*Contracaecum* sp.	+,5		+	+	+		+		+,1	+	+	+
*Goezia* sp.	5											5
*Hysterothylacium patagonense*	+,10,19			+,19	+,10,19							
*Hedruris suttonae*	+,5			+,5	+							
*Camallanus corderoi*	+,5,10		+	+	+,5,10					+		+
*Pseudodelphys limnicola*	+											
*Rhabdochona* sp.	5											
*Acanthocephalus tumescens*	+,5,10,14,20		5	+,5,10	+,5,10	5						
*Polymorphus* sp. 1										+		
*Polymorphus* sp. 2	+						+	+	+,1			
*Pomphorhynchus patagonicus*	+,5,21,22,23,24			+	+,5,21				+,1			

## 4 Discussion

The results of the present work show that three main groups can be distinguished in the helminthological fauna of alien fishes in Patagonia: 1) helminths co–introduced with exotic fish, which continue to parasitize the original alien hosts, 2) native larval helminths that are naturally distributed in Patagonia (mainly parasitizing fish–eating birds) and which currently parasitize the exotic fish of the area, and 3) adults of native helminth species that infect exotic fish.

The first group is mostly composed of monogeneans. Indeed, we recorded 13 monogenean species that were introduced with fish and continue to parasitize only their original hosts, like *D. anchoratus*, *D. extensus,* and *Pseudacolpenteron* sp., co–introduced with *C. carpio.* Monogeneans co–introduced with Brazilic fishes are the most diverse group, including *Diaphorocleidus* sp., *C.* cf. *costaricensis*, and a species of *Gyrodactylus* that arrived with the characid *C. interruptus*. The poecilid *C. decemmaculatus* introduced three species of *Gyrodactylus* and the callichthyid *C. paleatus* two gyrodactylids: *G. anysopharynx* and *G. superbus,* and the ancyrocephalid *P. platensis*. In addition, one species of *Gyrodactylus* arrived with the anablepid *J. multidentata*. All these monogeneans have established self–sustained-populations in Patagonia, and are still restricted to populations of the original, invasive, Brazilic hosts. Nowadays there are well–established populations of these Brazilic fishes in Patagonia, *C. interruptus*, and *C. paleatus* having mostly been accidentally introduced as adults in the 90's by aquarism and by using as bait (Peréz and López Cazorla, 2008). No data is available as to how *J. multidentata* and *C. decemmaculatus* were introduced; however, *J. multidentata* was first detected around 100 years ago in Colorado River (Baigún et al., 2002), and the presence of *C. decemmaculatus* was registered in the 60′s in Negro River (Ringuelet et al., 1967). The strict host specificity of the monogeneans provides an explanation for the lack of host–switching. Additionally, the absence of native fish species from the same families as the invading fish could contribute to the lack of co–invasions. Remarkably, only 6 species of monogeneans were previously recorded in the natives *G. maculatus* and *P. trucha* (Viozzi and Brugni, 2003; Viozzi et al., 2009; Fernández et al., 2010, 2015a; 2015b; Vega et al., 2011; Flores et al., 2016), therefore our present records represent rather rich component of monogenean species that have been introduced into Patagonian environments. One species which belongs to this first group, at least to date, is the cestode *S. acheilognathi*, which in Patagonia has been recorded only in the introduced common carp, *C. carpio.* This invasive cestode is originally from Asia, and has been co–introduced worldwide, mainly with carp. In South America this parasite was recorded in 5 freshwater fish species from Brazil (Alves et al., 2017). Asian carp have been reported in lakes from Buenos Aires Province since 1925 (Rosso, 2006), but reached Patagonian environments only recently, about 25 years ago (Peréz and López Cazorla, 2008). This parasite has the potential to be transferred to other fish species, thus becoming a potential threat to native freshwater fish species (Salgado–Maldonado and Pineda–López, 2003; Pérez–Ponce de Leon et al., 2017). In Argentina *S. achilognathi* is distributed in the rivers Neuquén, Limay and Negro, which constitute the southernmost limit of the known distribution of this parasite on the American Continent. Dams built on Neuquén and Limay rivers serve, for the moment, to contain the invasion of the fish upstream, although it is expected that they can overcome this obstacle, since the embryonated eggs or fingerlings could be transported by birds or by human activities. This possibility is a cause for concern, since the upper basins of the river include an important lake network located in protected areas of national parks.

The second group in the composition of helminth communities is composed of larval forms parasitizing alien fishes. Their definitive hosts are mainly fish–eating birds; for example, the metacercariae of *A. angrense*, *Diplostomum* sp., *Posthodiplostomum* sp., *Pygidiopsis* sp., *S. uruguayense* and *Tylodelphys* spp., as well as the nematode *Contracaecum* sp. These are generalist helminths, and their natural geographic distribution includes lakes and rivers in Patagonia (Ortubay et al., 1994; Viozzi et al., 2009; Fernández et al., 2012, 2015a; Flores et al., 2016). Fish are intermediate or paratenic hosts for these parasites and serve as trophic ways to reach the birds that are the definitive hosts. Helminths display reduced host specificity in fish, which increases their chances of reaching the definitive host (Esch et al., 1988). However, due to the large size of salmonids, it is not likely that adult fishes can be consumed by piscivorous birds, so in cases like diplostomids or *Contracaecum* sp., these hosts could represent a dead end for the parasites. The metacercariae of the digenean *A*. *apophalliformis*, which mature in ichthyophagous fishes, can be included in this second group. The occurrence of this helminth in alien fish is a natural consequence of their distribution in these Patagonian aquatic environments.

The third component of this helminthological fauna is the most interesting from our point of view, consisting of 15 native taxa that have been able to infect the invading fish, as adults. This group includes the monogenean *D. andinus*; trematodes *A. apophalliformis, A. patagonicum*, *Derogenes* sp., *D. patagonicus*, *S*. *macrophallus*, *S*. *szidati*, the cestodes *C*. *macdonaghi*, and *A*. *mirabilis*, the nematodes *H*. *patagonense*, *H*. *suttonae*, *C*. *corderoi*, and *P*. *limnicola*, and the acanthocephalans *A*. *tumescens* and *P*. *patagonicus*. Previous records of these species in native fishes (Ortubay et al., 1994; Viozzi et al., 2009; Fernández et al., 2012, 2015a; Flores et al., 2016) indicate that these helminth species are native and have developed in Patagonian ecosystems, and shown remarkable ability to invade new host species and establish reproductive populations in them. Eight of these species were found to be gravid in non–native fishes, including: *D*. *patagonicus*, *Derogenes* sp., *S*. *szidati*, *S*. *macrophallus*, *C*. *macdonaghi*, *C*. *corderoi*, *H*. *suttonae*, and *A*. *tumescens*. The presence of gravid parasites in alien fishes suggests the possibility of spillback, such that alien hosts amplify transmission, leading to a subsequent increase in infection of native hosts (Kelly et al., 2009). The relatively high abundance of these species in the helminth communities of native fish (Viozzi et al., 2009; Fernández et al., 2015a; Flores et al., 2016), lead us to suppose that their forms of transmission are abundant in aquatic environments. Once the species of exotic fish enter Patagonia they find a great abundance of these propagules, which acts in favor of the invasion and colonization of these native species of helminths on these new hosts. However, in Patagonia, the salmonid *O. mykiss* acts as a sink in many life cycles of adult parasites, like the monogenean *D. andinus*; the digeneans *A. apophalliformis* and *A. patagonicum*; the cestode *A. mirabilis*; the nematodes *C. corderoi*, *H. patagonense*, and *P. limnicola*; and the acanthocephalan *P. patagonicus*, since parasites do not reach sexual maturity in these hosts (present data; Trejo, 1992, 1994).

Salmonids and Brazilic fishes were the hosts more succeptible to be infected by native parasites. Salmonids were introduced into Patagonia more than 100 years ago as embryos (Macchi et al., 1999; Pascual et al., 2001; Baigún and Ferriz, 2003; Rosso, 2006; Amalfi, 2009), so these fishes were introduced without metazoan parasites. Nowadays they have become numerically dominant in Patagonian environments, being *O. mykiss* the fish with the widest distribution in the region (Baigún and Ferris, 2003; Cussac et al., 2016). The time elapsed since the colonization of the salmonids, together with their success in establishing themselves as invasive fish species, have favored their chances of being colonized by native parasites. The only introduced helminth parasites that currently infect native fishes are the cestodes *D. latus* and *D. dendriticus* (Ortubay et al., 1994; Semenas, 2006; Viozzi et al., 2009), which were not introduced by fish. It is currently accepted that *D. latus* arrived with migratory populations from Northern Europe who settled in Patagonia (Semenas, 2006), and while there is no conclusive information about the origin of *D. dendriticus*, it probably arrived from the Northern Hemisphere (Kuchta et al., 2013). Larvae of these cestodes follow the general behaviour of generalist opportunistic larvae, using the fishes as trophic channels to reach their definitive host. However, there is 1 non–helminth parasite, the invasive copepod *Lernaea cyprinacea* wich has been co–introduced with *C. carpio,* and can be considered co–invasive as it has been found in the native *P. trucha* (Waicheim et al., 2017).

Host specificity plays an important role in determining regional faunistic composition of helminth communities (Salgado–Maldonado et al., 2005), and one of the most obvious and intriguing features of parasitism is the pronounced conservatism in the range of hosts used (high host specificity). However while this view provides a mechanism for the evolution of existing interactions (i.e. species become increasingly well-adapted to one another) it provides no basis for understanding the origins of novel interactions (Agosta et al., 2010), as those produced by co-introductions and co-invasion of parasites. Some introduced fish parasites in Patagonia show high specificity and only parasitize a single fish species, such as the 15 monogeneans, the cestodes *Hepatoxilon* sp., and *S. acheilognathi*, the larval nematode *Goezia* sp. and one species of larval acanthocephalans of the genus *Polymorphus*. These parasites are those that we consider to be co-introduced and that have not yet switched to the native fish or to other introduced fish species in Patagonia. The rest of the helminths registered in alien fishes parasitizes more than one species of native or introduced fish, and is represented by species of all groups, except by monogeneans, which are usually highly specific parasites throughout the world (Roberts and Janovy, 2005). In studies carried out in México, the more generalistic fish parasites were digenean larvae and the larval nematode *Contracaecum* sp. (Salgado–Maldonado et al., 2005, 2011). In Patagonia, *Contracaecum* sp. was also the most generalistic parasite, which was found in nine alien fish species. The number of known hosts is not the ideal measure of a parasite's host-specificity. Parasites may show higher growth rates and fecundity on some hosts than on others, i.e. some hosts may be ‘better’ hosts than others (Rohde, 1991; Poulin, 1992). In our survey some larvae and adults helminths showed low specificity for the host species such as the digenean *Tylodelphys* sp. 2, the cestode *Dibothriocephalus* spp., the nematode *C. corderoi*, and the acanthocephalan *A. tumescens,* which parasitize more than 5 alien fishes. However, it is worth noting that some of the native generalist adult parasites do not reach sexual maturity in alien fishes, such is the case of the digeneans *A. apophalliformis* and *A. patagonicus*, the cestode *A. mirabilis*, the nematodes *H. patagonense* and *P. limnicola*, and the acanthocephalan *P. patagonicus.* On the other hand, the nematode *C. corderoi* that was found in 6 fish species can only mature in *O. bonariensis*. The adult native parasites that are able to reach sexual maturity in all its hosts are the digeneans *D. patagonicus, Derogenes* sp., *S. szidati* and *S. macrophallus*, the cestode *C. macdonaghi*, the nematodes *Rhabdochona* sp. and *H. suttonae,* and the acanthocephalan *A. tumescens*.

This is the first review of the parasites of alien fishes in Argentina. To prepare this inventory we sampled most species of fish known to have been introduced into Patagonian freshwater basins, with the exception of five species with a narrow distribution in Patagonia: *A. eigenmanniorum*, *A. pampa*, *O. jenynsii*, *J. alternimaculata*, and *C. lacustris*. In total, the present review contains 12 new records of parasites for Argentina, 6 new records of parasites for Patagonia, and 29 new host–parasite records (Table 2). Nevertheless, this list is far for complete, taking into account that some southern large basins remain unexplored for parasites, like those of the rivers Chubut, Santa Cruz, and Grande, among others. Moreover, only very few specimens of some fish species have been examined, such as *S. namaycush* (only a few specimens), *O. tshawytscha* (1 specimen), *S. salar* (6), and *O. bonariensis* (9). Thus, future studies can be expected to increase the list of helminth species in different hosts of Argentinean Patagonia.

Patagonia has a depauperate fish fauna, so the limited availability of suitable hosts may decrease the opportunities for colonization of new hosts by parasites (Poulin, 1992). It is also probable that the environmental characteristics of Patagonia do not represent optimal conditions for species of introduced parasites to thrive; for example, almost all lakes and reservoirs with *C. carpio* in Patagonia lie below the thermal range of growth and preference for the species (Crichigno et al., 2016), thus parasites would also experience some kind of restriction concerning abiotic conditions. In the same way as Northern Patagonia represents the potential southern limit for some fish species like characids, anablepids, poecilids, and callichthyids, the abiotic conditions of the area would also constitute a limit for their parasites. However, climate change may generate new environmental scenarios that favor the establishment of alien fishes and their parasites.

## 5 Conclusions

In Patagonian alien fishes we found 43 parasite taxa (15 digeneans, 14 monogeneans, 5 cestodes, 5 nematodes, and 4 acanthocephalans), belonging to 22 families. Our data indicate that invading fish in Patagonia have transmitted fewer parasite species than they have received by spillback. In this aspect, exotic salmonids stand out as hosts which are highly susceptible to infection by parasites of Patagonian native fish. The salmonids *O. mykiss* (17 parasite taxa in total, and 15 native) and *S. fontinalis* (12 parasite taxa in total, and 11 native) were the fish species with the highest parasite species richness recorded. We acknowledge that the high numbers of helminths we report in salmonids in this work could be related to sampling bias, due to the high number of localities and fish examined compared to other fish families, as well as the wider distribution of salmonids in Patagonia. However, these records constitute very strong empirical evidence of the susceptibility of these salmonid species to infection by parasites of the native Patagonian fishes. Although the exotic fish of the Brazilic component also show certain susceptibility to native Patagonian helminths, as in the case of *J. multidentata*.

## Appendix A Supplementary data

Rauque et al Rauque et al
