# Supplementary material for: Helminth parasites of alien freshwater fishes in Patagonia (Argentina)
Source: Int J Parasitol Parasites Wildl. 2018 Oct 1;7(3):369–79. doi: 10.1016/j.ijppaw.2018.09.008 (PMC6174270; doi:10.1016/j.ijppaw.2018.09.008)
Supplement: Rauque et al [file mmc1.docx]

**Supplementary data**

Acronyms of collections for deposited helminth voucher specimens are: National Collection of Parasitology, Museo Argentino de Ciencias Naturales Bernardino Rivadavia, Buenos Aires, Argentina (MACN–Pa), Helmintological Collection of Museo de La Plata, La Plata, Argentina (MLP–He), Parasitological Collection of Universidad Nacional del Comahue, Bariloche, Argentina (UNCo–Pa), United States National Parasite Collection, Beltsville, Maryland, USA (USNPC), National Science Museum, Tokyo (NSMT), Helminthological Collection, Institute of Parasitology, Academy of Sciences of the Czech Republic, České Budějovice, Czech Republic (IPCAS), Collections of the Natural History Museum, London, United Kingdom (BMNH), Natural History Museum, Geneve, Switzerland (MHNG INVE), and Meguro Parasitological Museum, Tokyo (MPM).

**Parasite–host list**

**Monogenoidea**

**Family: Dactylogyridae Bychowsky, 1933**

#*Dactylogyrus anchoratus* Dujardin, 1845

**Hosts and localities:** *Cyprinus carpio* – Neuquén River (downstream Ballester Dam); Limay River (China Muerta Town); Negro River (Allen City).

**Site of infection:** gills.

**Stage:** gravid adults.

**Prevalence (P) and mean intensity (MI)**: downstream Ballester Dam (N=55, P=18%, MI=1.8); China Muerta Town (N=2, P=2/2, MI=4.5); Allen City (N=52, P=35%, MI=5.8).

**Origin:** co–introduced with *C. carpio*.

**Date of first detection in Patagonia:** June 2015 (present survey).

**Vouchers:** MACN–Pa 653/1 (present survey).

**Remarks:** *D*. *anchoratus* is a very commnon monogenean in Asian carp, already co–introduced with this host into many places, including Europe, North America and Mexico (Hoffman, 1999; Özer, 2002; Salgado–Maldonado, 2006). *Cyprinus carpio* is the only host recorded for *D. anchoratus.* Due to the high infection observed, wide distribution, and occurrence in several samples from different years (Table 1), it can be inferred that populations of this monogenean species are established in Patagonia.

*Dactylogyrus extensus* Mueller and Van Cleave, 1932

**Hosts and localities :** *Cyprinus carpio* – Colorado River (Casa de Piedra Reservoir), Neuquén River (downstream Ballester Dam), Limay River (downstream Arroyito Dam, China Muerta Town, Herradura Backwater), Negro River (Allen City, Guardia Mitre Town).

**Site of infection:** gills.

**Stage:** gravid adults.

**Prevalence (P) and mean intensity (MI)**: Casa de Piedra Reservoir (N=3, P=1/3, MI=31); downstream Ballester Dam (N=55, P=91%, MI=29); downstream Arroyito Dam (N=22, P=73%, MI=20.2); China Muerta Town (N=2, P=2/2, MI=14); Herradura Backwater (N=10, P=80%, MI=168.6), Allen City (N=52, P=90%, MI=23.2); Guardia Mitre Town(N=61, P=56%, MI=13.4).

**Origin:** co–introduced with *C. carpio.*

**Date of first detection in Patagonia:** December 2011 (present survey).

**Other records in Patagonia:** Waicheim et al*.* (2014) in *C. carpio*.

**Vouchers:** MACN–Pa 559/1–2 (Waicheim et al*.*, 2014), MACN–Pa 654/1 (present survey).

**Remarks:** *D*. *extensus* is a very common monogenean in Asian carp, already co–introduced with this host into many places, including Europe, North America and Mexico (Hoffman, 1999; Özer, 2002; Salgado–Maldonado, 2006). *Cyprinus carpio* is the only host recorded for *D. extensus.* Due to the high values of infection observed, wide distribution, and occurrence in several samples from different years (Table 1), it can be inferred that populations of this monogenean species are established in Patagonia*.*

*Duplaccessorius andinus* Viozzi and Brugni, 2004

**Hosts and localities:** †*Oncorhynchus mykiss* – Senguer River (Musters Lake).

**Site of infection:** gills.

**Stage:** immature adults.

**Origin:** native.

**Other records in Patagonia:** Viozzi and Brugni (2004) in *Percichthys trucha*.

**Vouchers:** MACN–Pa 425/1–7, MLP–He 5296–5300, UNCo–Pa 186/1–8, 187/1, USNPC 94369–94373 (Viozzi and Brugni 2004), MACN–Pa 649/1 (present survey).

**Remarks:** *D*. *andinus* is a common parasite in populations of the native *P. trucha. Oncorhynchus mykiss* is probably an accidental host because a single infected fish was recorded here, from 14 examined in Musters Lake.

**Philocorydoras platensis* Suriano, 1986

**Hosts and localities:** *Corydoras paleatus* – Neuquén River (Arroyón Stream).

**Site of infection:** gills.

**Stage:** gravid adults.

**Prevalence (P) and mean intensity (MI)**: Arroyón Stream (N=13, P=15%, MI=1.5).

**Origin:** co–introduced with *C. paleatus*.

**Date of first detection in Patagonia:** October 2014 (present survey).

**Vouchers:** MACN–Pa 642/1 (present survey).

**Remarks:** *Philocorydoras platensis* was first described from *C. paleatus* by Suriano (1986) in an Argentinean shallow lake located in the Subtropical Potamic Icthyogeographical Ecoregion (Cussac et al*.*, 2009). *Corydoras paleatus* is the only host recorded for *P. platensis* in Patagonia, with low parasite infection values*.*

*Pseudacolpenteron* sp.

**Hosts and localities:** *Cyprinus carpio* – Colorado River (Casa de Piedra Reservoir), Neuquén River (downstream Ballester Dam), Limay River (downstream Arroyito Dam, China Muerta Town), Negro River (Allen City, Guardia Mitre Town).

**Site of infection:** canals of the scales along the somatic lateral line.

**Stage:** gravid adults.

**Prevalence (P) and mean intensity (MI)**: Casa de Piedra Reservoir (N=3, P=2/3, MI=3.5); downstream Ballester Dam (N=55, P=24%, MI=3.2); downstream Arroyito Dam (N=22, P=4.5%, MI=1); China Muerta Town (N=2, P=2/2%, MI=7); Allen City (N=52, P=33%, MI=2.3); Guardia Mitre Town (N=61, P=16%, MI=3).

**Origin:** co–introduced with *C. carpio*.

**Date of first detection in Patagonia:** November 2011 (data of the present survey).

**Other records in Patagonia:** Waicheim et al*.* (2014) in *C. carpio*.

**Vouchers**: MACN–Pa 560/1–2 (Waicheim et al*.*, 2014), MACN–Pa 652 (present survey).

**Remarks:** species of *Pseudacolpenteron* have been cited from the gills and fins of cyprinids in Europe and North America (Yamaguti, 1963; Rogers, 1968). While the validity of the genus *Pseudacolpenteron* has been controversial (Yamaguti, 1963; Rogers, 1968), it seems that sites of infection within the host (the ureters and kidney for *Acolpenteron* spp., and gills and buccal cavity for *Pseudacolpenteron* spp.) represent diagnostic features of the 2 genera (Fayton and Kristky, 2013). Waicheim et al*.* (2014) indicated the presence of *Pseudacolpenteron* sp. in the lateral line system of fishes from Patagonia, which represents a new site of infection for members of this genus. The species reported here resembles *P*. *pavlovskii* Bychowsky and Gussev, 1955 (Molnár, 2012); however, additional morphological comparative studies are needed to determine whether the difference in infection indicates the presence of a different spapophalecies. The infection values, wide distribution, and occurrence in several samples from different years (Table 1) indicate that populations of this monogenean species are established in Patagonia.

*#Diapharocleidus* sp.

**Hosts and localities:** *Cheirodon interuptus* – Neuquén River (Arroyón), Valcheta Stream (Chipauquil Town).

**Site of infection:** gills.

**Stage:** gravid adults.

**Prevalence (P) and mean intensity (MI)**: Arroyón (N=37, P=50%, MI=2); Chipauquil Town (N=15, P=39%, MI=1.5).

**Origin:** co–introduced with *C. interruptus*.

**Date of first detection in Patagonia:** October 2009 (present survey).

**Vouchers**: MACN–Pa 650/1 (present survey).

**Remarks:** Seven species of the genus *Diapharocleidus* have been recorded from Brazil, México, Panamá, and Trinidad for eight host species (Jogunoori et al., 2004; Moreira et al., 2016; Da Silva Silveira De Almeida and Cohen, 2011; Acosta et al., 2013; Mendoza–Franco et al., 2007, 2009). However, the specimens from *C. interruptus* of Patagonia seem to be a different species, deserving additional morphological studies. The infection values, wide distribution, and occurrence in several samples from different years (Table 1) indicate that populations of this monogenean species are established in Patagonia.

*#Characithecium* cf. *costaricensis* (Price and Bussing, 1967)

**Hosts and localities:** *Cheirodon interuptus –* Neuquén River (Arroyón), Valcheta Stream (Chipauquil Town).

**Site of infection:** gills.

**Stage:** gravid adults.

**Overall prevalence (P) and mean intensity (MI)**: Arroyón (N=37, P=38%, MI=17.6); Chipauquil Town (N=15, P=80%, MI=10.3).

**Origin:** co–introduced with *C. interruptus*.

**Date of first detection in Patagonia:** October 2010 (present survey).

**Vouchers**: MACN–Pa 651/1 (present survey).

**Remarks:** The morphology and measurements of the present specimens agree with *Characithecium costaricensis* as described by Mendoza–Franco et al. (2009)*.* This species has been recorded in characids from Mexico to Brazil (Mendoza–Franco et al*.*, 2009; Acosta et al*.*, 2015). In Argentina other species of the genus have been cited in *O. jenynsii* (Rossin and Timi, 2014). Therefore, the host record and geographic distribution of this species has now been extended southward. Infection values, wide distribution, and occurrence in several samples from different years (Table 1) indicate that populations of this monogenean species are established in Patagonia.

**Family: Gyrodactylidae Van Beneden and Hesse, 1863**

*#Gyrodactylus anisopharynx* Popazoglo and Boeger, 2000

**Hosts and localities:** *Corydoras paleatus* – Neuquén River (Arroyón Stream).

**Site of infection:** tegument.

**Stage:** gravid adults.

**Prevalence (P) and mean intensity (MI)**: Arroyón Stream (N=13, P=31.3%, MI=1.4).

**Origin:** co–introduced with *C. paleatus*.

**Date of first detection in Patagonia:** March 2014 (present survey).

**Vouchers:** MACN–Pa 643/1 (present survey).

**Remarks:** Popazoglo and Boeger (2000) described two distinct variants of *Gyrodactylus anisopharynx,* named “large–pharynx” and “small–pharynx”, on the basis of the morphology of the distal and proximal pharyngeal bulb. They were found on the sympatric hosts *Corydoras paleatus* and *Corydoras ehrhardti*, from the rivers of Curitiba (Paraná, Brazil). Bueno–Silva and Boeger (2009) later discriminated the forms “large–pharynx” and “small–pharynx” by morphometric analysis of their hard parts, and proposed two independent species: *G. anisopharyx* for “large–pharynx” and *G. corydori* for “small–pharynx”. Measurements of the pharynx and the haptoral sclerites enables the specimens found in *Corydoras paleatus* from Patagonia to be identified as *G. anisopharynx*. High infection values indicate that populations of this monogenean species are established in Patagonia.

**Gyrodactylus superbus* (Szidat, 1973)

**Syn.:** *Paragyrodactylus superbus* Szidat, 1973

**Hosts and localities:** *Corydoras paleatus* – Neuquén River (Arroyón Stream).

**Site of infection:** tegument.

**Stage:** gravid adults.

**Prevalence (P) and mean intensity (MI)**: Arroyón Stream (N=13, P=71.4%, MI=2.7).

**Origin:** co–introduced with *C. paleatus*.

**Date of first detection in Patagonia**: March 2014 (present survey).

**Vouchers:** MACN–Pa 644/1.

**Remarks**: Szidat (1973) described *Paragyrodactylus superbus* from *C. paleatus* from an aquarium at the Museo de Ciencias Naturales “Bernardino Rivadavia”, Buenos Aires, Argentina. Popazoglo and Boeger (2000) later redescribed specimens collected from rivers of the Metropolitan Area of Curitiba (Paraná, Brazil), and transferred the species to the genus *Gyrodactylus.* Infection values, wide distribution, and occurrence in several samples from different years (Table 1), indicate that populations of this monogenean species are established in Patagonia.

The next five species of *Gyrodactylus* were found parasitizing examined alien fishes. Morphological comparisons suggest these are different species which have not been previously recorded in Patagonian freshwater environments. They can be distinguished from the other gyrodactylids from Patagonia by a combination of features, including the Male Copulatory Organ (MCO) spinelet number, the superficial bar shape, and characteristics of the superficial anchor root, among others. In Patagonia there is a record of *Gyrodactylus* sp. in *Gymnocharacinus bergi* from Valcheta Stream (Ortubay et al*.*, 1994).

*#Gyrodactylus* sp. 1

**Hosts and localities:** *Jenynsia multidentata –* Colorado River (Casa de Piedra Reservoir), Neuquén River (Arroyón Stream, downstream Ballester Dam), Limay River (downstream Arroyito Dam, Plottier City, Gatica Beach).

**Site of infection:** tegument.

**Stage:** gravid adults.

**Prevalence (P) and mean intensity (MI):** Casa de Piedra Reservoir (N=44, P=59%, MI=7); Arroyón Stream (N=90, P=3%, MI=2); downstream Ballester Dam (N=22, P=100%, MI=3.3); downstream Arroyito Dam (N=3, P=100%, MI=12), Plottier City (N=5, P=100%, MI=13), Gatica Beach (N=2, P=2/2, MI=4.5).

**Origin:** co–introduced with *J. multidentata*.

**Date of first detection in Patagonia:** May 2012 (present survey).

**Vouchers:** MACN–Pa 645/1 (present survey).

**Remarks:** Infection values, wide distribution, and occurrence in several samples from different years (Table 1), indicate that populations of this monogenean species are established in Patagonia.

*#Gyrodactylus* sp. 2

**Hosts and localities:** *Cheirodon interuptus –* Neuquén River (Arroyón stream).

**Site of infection:** fins.

**Stage:** gravid adults.

**Prevalence (P) and mean intensity (MI):** Arroyón Stream (N=37, P=60%, MI=2.8).

**Origin:** co–introduced with *C. interruptus*.

**Date of first detection in Patagonia:** March 2013 (present survey).

**Vouchers:** MACN–Pa 646/1 (present survey).

**Remarks:** Infection values, wide distribution, and occurrence in several samples from different years (Table 1), indicate that populations of this monogenean species are established in Patagonia.

*#Gyrodactylus* sp. 3

**Hosts and localities:** *Cnesterodon decemmaculatus –* Neuquén River (Arroyón Stream, downstream Ballester Dam).

**Site of infection:** tegument.

**Stage:** gravid adults.

**Overall prevalence (P) and mean intensity (MI):** Arroyón Stream (N=12, P=33%, MI=1.5); downstream Ballester Dam (N=14, P=87%, MI=4.5).

**Origin:** co–introduced with *C. decemmaculatus*.

**Date of first detection in Patagonia:** March 2014 (present survey).

**Vouchers:** MACN–Pa 647/1 (present survey).

**Remarks:** Infection values, wide distribution, and occurrence in several samples from different years (Table 1), indicate that populations of this monogenean species are established in Patagonia.

*#Gyrodactylus* sp. 4

**Hosts and localities:** *Cnesterodon decemmaculatus –* Neuquén River (Arroyón Stream, downstream Ballester Dam).

**Site of infection:** tegument.

**Stage:** gravid adults.

**Prevalence (P) and mean intensity (MI):** Arroyón Stream (N=12, P=17%, MI=2.6); downstream Ballester Dam (N=14, P=28%, MI=1.6).

**Origin:** co–introduced with *C. decemmaculatus.*

**Date of first detection in Patagonia:** March 2014 (present survey).

**Vouchers:** MACN–Pa 648/1 (present survey).

**Remarks:** Infection values, wide distribution, and occurrence in several samples from different years (Table 1), indicate that populations of this monogenean species are established in Patagonia.

*#Gyrodactylus* sp. 5

**Hosts and localities:** *Cnesterodon decemmaculatus –* Neuquén River (downstream Ballester Dam).

**Site of infection:** tegument.

**Stage:** gravid adults.

**Prevalence (P) and mean intensity (MI):** downstream Ballester Dam (N=14, P=1.6%, MI=3).

**Origin:** co–introduced with *C. decemmaculatus*.

**Date of first detection in Patagonia:** March 2014 (present survey).

**Vouchers:** the small amount of worms and/or the state of conservation did not allow the preparation of voucher specimens from the present survey.

**Remarks:** Infection values, wide distribution, and occurrence in several samples from different years (Table 1), indicate that populations of this monogenean species are established in Patagonia.

**Digenea**

**Family: Allocreadiidae Looss, 1902**

*Allocreadium patagonicum* Shimazu, Urawa and Coria, 2000

**Syn.:** *Polylekithum percai* Ostrowski de Nuñez, Brugni and Viozzi, 2000.

**Hosts and localities:** †*Oncorhynchus mykiss –* Puelo River (Epuyén Lake).

**Site of infection:** intestine.

**Stage:** immature adults.

**Origin:** native.

**Other records in Patagonia:** Ostrowski de Nuñez et al*.* (2000), Shimazu et al*.* (2000), and Rauque et al*.* (2003) in *Percichthys trucha*.

**Vouchers:** MANC–Pa 394/1–2, UNCo–Pa 107/1–18, BMNH 1999.10.19.1 (Ostrowski de Nuñez et al*.* 2000), NSMT–Pl 4583, IPCAS D–424 (Shimazu et al*.* 2000), the small amount of worms and/or the state of conservation did not allow the preparation of voucher specimens from the present survey.

**Remarks:** *Allocreadium patagonicum* is generally restricted to *P. trucha* from Andean Lakes of Patagonia. In salmonids it was only recorded in Epuyén Lake, with low prevalence values (11%). *Oncorhynchus mykiss* probably represents a sink for this parasite. Flores et al*.* (2004) proposed that *A. patagonicum* and *P. percai* are synonyms.

### Family: Derogenidae Nicoll, 1910

*Deropegus patagonicus* (Szidat, 1956)

**Syn.:** *Genarches patagonicus* Szidat, 1956, *Derogenes patagonicus* Yamaguti, 1971, *Deropegus patagonicus* Shimazu, Urawa and Coria, 2000, *Thometrema patagonica* Lunaschi and Drago, 2000.

**Hosts and localities:** †*Jenynsia multidentata –* Neuquén River (Arroyón stream, downstream Ballester Dam).

**Site of infection:** stomach.

**Stage:** gravid adults.

**Origin:** native.

**Other records in Patagonia:** Szidat (1956), and Lunaschi and Drago (2000) in *Percichthys trucha*, Shimazu et al*.* (2000) in *Oncorhynchus mykiss*, *Salmo trutta*, *Salvelinus fontinalis*, and *P. trucha*, and Ostrowski de Núñez et al*.* (2017) in *S. trutta*, *S. fontinalis*, and *P. trucha*.

**Vouchers:** NSMT–Pl 4584–4587, IPCAS D–423 (Shimazu et al*.*, 2000), the small amount of worms and/or the state of conservation did not allow the preparation of voucher specimens from the present survey.

**Remarks:** Szidat (1956) described *Genarches patagonicus* in *P. trucha* from Pellegrini Lake. Yamaguti (1971) transferred the species to *Derogenes patagonicus*. Lunaschi and Drago (2000) redescribed the species from Pellegrini Lake and transferred it to *Thometrema patagonica*, while simultaneously Shimazu et al*.* (2000) transferred it to *Deropegus patagonicus* for specimens from Aluminé Lake. This species is restricted to the North of Argentinean Patagonia. *Percichthys trucha* is the original host and this salmonid–infecting parasite seems to be a case of spillback. *Jenynsia multidentata* would be an accidental host, since only 1 parasite was found.

*Derogenes* sp.

**Hosts and localities:** †*Oncorhynchus mykiss –* Limay River (Escondido Lake, Morenito Lake, Moreno Lake, Gutiérrez Lake, Nahuel Huapi Lake, Alicura Reservoir), Puelo River (Puelo Lake).

†*Salmo salar* – Limay River (Alicura Reservoir).

†*Salmo trutta* – Limay River (Moreno Lake, Nahuel Huapi Lake, Alicura Reservoir, Piedra del Águila Reservoir).

†*Salvelinus fontinalis* – Limay River (Moreno Lake).

**Site of infection:** stomach.

**Stage:** gravid adults.

**Origin:** native.

**Other records in Patagonia:** Fernández et al*.* (2015a) in *Galaxias maculatus.*

**Vouchers:** MACN–Pa 637/1, 638/1 (present survey).

**Remarks:** Specimens collected in the present survey have a post–equatorial ventral sucker, rudimentary Joel organ, seminal receptacle in uterus, globular sinus sac, and lack of filaments or spines in the eggs, characteristics which allow it to be included in the genus *Derogenes* sp. following Gibson (2002); however, this could be a new species. This species is restricted to fishes from Andean Patagonian Lakes in Argentina. *Galaxias maculatus* seems to be the original host, and their presence in salmonids represents a case of spillback, probably acquired by post–cyclic transmission.

**Family: Diplostomidae Poirier, 1886**

*Austrodiplostomum mordax* Szidat and Nani, 1951

**Site of infection:** brain.

**Stage:** metacercaria.

**Origin:** native.

**Other records in Patagonia:** Szidat and Nani (1951, 1952) in *Odontesthes hatcheri*, Ortubay et al*.* (1989) in *Oncorhynchus mykiss*, Ortubay et al*.* (1994) in *O. mykiss*, *Aplochiton teniatus*, *G. maculatus*, *Galaxias platei*, *Jenynsia multidentata*, *Odontethes hatcheri* and *Percichthys trucha*, Ruiz (2007) in *O. hatcheri*, Flores et al*.* (2016) in *Odontesthes bonariensis* and *O. hatcheri*.

**Vouchers:** MLP–He 7136 (Flores et al*.*, 2016).

**Remarks:** Metacercariae of *A. mordax* parasitize freshwater fishes from Buenos Aires and Córdoba Provinces, and from Argentinean Patagonia (Szidat and Nani, 1951, 1952; Fuster de Plaza and Boschi, 1957; Ortubay et al*.*, 1989, 1994, Flores et al*.*, 2016; Ostrowski de Núñez, 2017). This parasite uses fish–eating birds as its definitive host (Ostrowski de Núñez, 1968), so it has a wide distribution in Argentina. Szidat and Nani (1951) and Fuster de Plaza and Boschi (1957) cited *Diplostomulum mordax*. Host cited as *Jenynsia lineata* by Ortubay et al*.* (1994). This species was not found in the present survey.

*Diplostomum* sp.

**Hosts and localities:** *Oncorhynchus mykiss –* Limay River (Gutiérrez Lake), Senguer River (Musters Lake), Azopardo River (Escondido Lake, Margarita Lake).

*Salvelinus fontinalis* – Limay River (Gutiérrez Lake).

**Site of infection:** lens.

**Stage:** metacercaria.

**Origin:** native.

**Other records in Patagonia:** Ortubay et al*.* (1994) in *O. mykiss*, *Salmo trutta*, *S. fontinalis*, *Salvelinus namaycush*, *Aplochiton zebra*, *Galaxias maculatus*, *Galaxias platei*, *Odontesthes hatcheri*, and *Percichthys trucha*, Semenas (1998) in *O. mykiss*, Viozzi et al*.* (2009) in *G. maculatus*, Fernández et al*.* (2012) in *A. zebra*, and Flores et al*.* (2016) in *O. hatcheri*.

**Vouchers:** MACN–Pa 454/1–2 (Viozzi et al*.* 2009), MACN–Pa 525 (Fernández et al*.* 2012).

**Remarks:** Ortubay et al*.* (1989) reported the presence of *Diplostomum minutum* Szidat, 1964 metacercariae. This record needs to be revised. It is probably a complex of species.

*Posthodiplostomum* sp.

**Hosts and localities:** *Jenynsia multidentata –* Neuquén River (Arroyón Stream, downstream Ballester Dam).

**Site of infection:** abdominal cavity.

**Stage:** metacercaria.

**Origin:** native.

**Other records in Patagonia:** Ortubay et al*.* (1994), Revenga et al*.* (2005), Viozzi et al*.* (2009), Ritossa et al*.* (2013, 2014), and Fernández et al*.* (2010, 2015a, 2015b) in *Galaxias maculatus*.

**Vouchers:** MACN–Pa 452/1–2 (Viozzi et al. 2009), MACN–Pa 558/5–7, UNCo–Pa 241/1–12 (Ritossa et al. 2013).

**Remarks:** Doma and Ostrowski de Núñez (1994) recorded the presence of *Posthodiplostomum namum* Dubois, 1937 in *J. multidentata* and *Cnesterodon decemmaculatus* from Chascomús Lake in Buenos Aires Province. Our specimens differ morphologically from other known species in Patagonia.

**Tylodelphys* sp. 1

**Hosts and localities:** *Odontesthes bonariensis –* Neuquén River (Pellegrini Lake).

**Site of infection:** gall bladder.

**Stage:** metacercaria.

**Origin:** native?

**Remarks:** This metacercaria was found only in the alien *O. bonariensis* from Pellegrini Lake. It is likely that this parasite can also be found in the gall bladder of the native silverside *Odontesthes hatcheri*.

*Tylodelphys* sp. 2

**Hosts and localities:** *Oncorhynchus mykiss –* Limay River (Moreno Lake, Gutiérrez Lake, Nahuel Huapi Lake, Alicura Reservoir, downstream Arroyito Reservoir).

†*Salmo trutta* – Limay River (Nahuel Huapi Lake).

†*Salvelinus fontinalis* – Limay River (Moreno Lake, Gutiérrez Lake, Nahuel Huapi Lake).

**Site of infection:** eye humour, brain.

**Stage:** metacercaria.

**Origin:** native.

**Other records in Patagonia:** Szidat and Nani (1951) in *Jenynsia multidentata*, Ortubay et al. (1989) in *O. mykiss*, *Galaxias platei*, and *Odontesthes hatcheri*, Ortubay et al. (1994) in *O. mykiss*, *Aplochiton zebra*, *Galaxias maculatus*, *G. platei*, *O. hatcheri*, *O. bonariensis*, *J. multidentata* and *Percichthys trucha*, Quaggiotto and Valverde (1992), Flores (1997), Flores and Baccalá (1998), Revenga and Scheinert (1999), Flores and Semenas (2002), Revenga et al*.* (2005), and Viozzi et al*.* (2009) and Fernández et al*.* (2010, 2015a, 2015b) in *G. maculatus*, and Fernández et al*.* (2012) in *A. zebra*, and Szidat and Nani (1951), Viozzi and Flores (2002), and Flores et al*.* (2016) in *Odontesthes hatcheri*.

**Vouchers:** MACN–Pa 368 (Quaggiotto and Valverde 1992), MACN–Pa 452/1–2 (Viozzi et al*.* 2009), MACN–Pa 526 (Fernández et al*.* 2012), MLP–He 7138 (Flores et al*.* 2016).

**Remarks:** Metacercariae of *Tylodelphys argentinus* Quaggiotto and Valverde, 1992, *Tylodelphys barilochensis* Quaggiotto and Valverde, 1992, *Tylodelphys crubensis* Quaggiotto and Valverde, 1992, *Tylodelphys destructor* Szidat and Nani, 1951 and *Tylodelphys cardiophilus* Szidat, 1969 have been recorded in Patagonia (Quaggiotto and Valverde, 1992; Flores, 1997; Flores and Baccalá, 1998; Revenga and Scheinert, 1999; Flores and Semenas, 2002; Revenga et al*.*, 2005; Flores et al*.*, 2016; Szidat and Nani, 1951; Viozzi and Flores, 2002; Ortubay et al., 1994; Flores et al*.*, 2016). However, Blasco–Costa et al*.* (2017) considered all these to be *incertae sedis*. They probably represent a complex of species.

**Family: Echinostomatidae Looss, 1899**

*Stephanoprora uruguayense* Holcman–Spector and Olagüe, 1989

**Hosts and localities:** †*Cheirodon interruptus* – Neuquén River (Arroyón Stream).

†*Cnesterodon decemmaculatus* – Neuquén River (Arroyón Stream, downstream Ballester Dam), Valcheta stream (Chipauquil).

†*Jenynsia multidentata –* Neuquén River (Arroyón Stream, downstream Ballester Dam).

**Site of infection:** gills.

**Stage:** metacercaria.

**Origin:** native.

**Other records in Patagonia:** Ostrowski de Núñez et al*.* (2004), Viozzi et al*.* (2009), and Fernández et al*.* (2010, 2015a, 2015b) in *Galaxias maculatus*, Fernández et al*.* (2012) in *Aplochiton zebra*, and Flores et al*.* (2016) in *Odontesthes hatcheri*.

**Vouchers:** MACN–Pa 455 (Viozzi et al*.*, 2009).

**Remarks**: *Stephanoprora uruguayense* was originally described parasitizing *Larus dominicanus* from Montevideo, Uruguay (Holcman–Spector and Olagüe, 1989). In Patagonia this parasite is commonly found in the native fishes, with high infection (Ostrowski de Núñez et al*.*, 2004; Viozzi et al*.*, 2009; Fernández et al*.*, 2010, 2012, 2015a, 2015b; and Flores et al*.*, 2016). This parasite uses fish–eating birds as its definitive host (Ostrowski de Núñez et al*.*, 2004), so has a wide distribution in Argentina.

**Family: Heterophyidae Leiper, 1909**

**Ascocotyle* cf. *angrense* Travassos, 1916

**Hosts and localities:** †*Odontesthes bonariensis* – Neuquén River (Pellegrini Lake).

**Site of infection:** gills.

**Stage:** metacercaria.

**Origin:** native?

**Remarks:** *A. angrense* was recorded in Cyprinodontiformes near La Plata River in Buenos Aires Province (Ostrowski de Núñez, 1974). In Patagonia, no fishes have been recorded as infected with species of *Ascocotyle* in gills. *Ascocotyle* species are parasites of fish–eating birds (Drago and Lunaschi, 2011), so the species cited here probably have a wide distribution range in Argentina.

**Ascocotyle* cf. *diminuta* Stunkard and Haviland, 1924

**Hosts and localities:** *Jenynsia multidentata* – Neuquén River (Arroyón Stream, downstream Ballester Dam).

**Site of infection:** gills.

**Stage:** metacercariae.

**Origin:** unknown.

**Remarks:** This parasite was recorded in several fishes from Buenos Aires Province: *J. multidentata*, *Cnesterodon decemmaculatus*, *Cychlasoma fasciatus* and *Odontesthes bonariensis,* and also in *Gambusia affinis* (Ostrowski de Núñez, 1993; Drago, 2012). In Patagonia, no fishes have been recorded as infected with species of *Ascocotyle* in gills, but in the present study it was found in fishes that were introduced as adults. Therefore, we cannot define whether it was introduced with this fish or its distribution is wider since it is a parasite of fish–eating birds.

**Ascocotyle* cf. *tertia* Ostrowski de Núñez, 2001.

**Hosts and localities:** †*Odontesthes bonariensis* – Neuquén River (Pellegrini Lake).

*Jenynsia multidentata –* Neuquén River (Arroyón Stream, downstream Ballester Dam).

**Site of infection:** bulbus arteriosus.

**Stage:** metacercaria.

**Origin:** unknown.

**Remarks:** In fishes from Argentina, *A. tertia* was recorded in *J. multidentata*, *Cnesterodon decemmaculatus* and in the introduced *Gambusia affinis* from Buenos Aires Province (Ostrowski de Núñez, 2001). In Patagonia no native fishes have been recorded as infected with this parasite. Ortubay et al*.* (1994) recorded the presence of *Ascocotyle* sp. in the heart of *Odontesthes hatcheri* from Pellegrini Lake (Río Negro Province), Drago (2012) and Flores et al*.* (2016) recorded *Ascocotyle* sp. in the heart of *O. bonariensis* from shallow lakes of Buenos Aires Province. *Ascocotyle* species of are parasites of fish–eating birds (Drago and Lunaschi, 2011), so the species cited here probably have a wide distribution in Argentina or were introduced by *J. multidentata*.

*Pygidiopsis* sp.

**Hosts and localities:** *Jenynsia multidentata –* Neuquén River (Arroyón Stream).

†*Odontesthes bonariensis –* Neuquén River (Pellegrini Lake).

**Site of infection:** intestinal wall.

**Stage:** metacercaria.

**Origin:** native.

**Other records in Patagonia:** Ortubay et al*.* (1994) in *Odontesthes hatcheri*.

**Remarks:** In Argentina, 4 species of the genus have been reported as infecting wild fishes of Buenos Aires Province: *Pygidiopsis australis* Ostrowski de Núñez, 1996 in *Cnesterodon decemmaculatus*, *Pygidiopsis crassus* Ostrowski de Núñez, 1995 in *C. decemmaculatus* and *Jenynsia multidentata*, *Pygidiopsis pindoramensis* Travassos, 1929 in *C. decemmaculatus* and *Phalloceros caudimaculatus* (Ostrowski de Núñez, 1974, 1976) and *Pygidiopsis* sp. in *P. caudimaculatus* and *C. decemmaculatus* (Lunaschi et al*.*, 2007). In Patagonia, specimens of this genus are restricted to Neuquén River Basin where they were also found in the native *O. hatcheri* (Ortubay et al*.*, 1994). This parasite shows important morphological differences compared to the known species. *Pygidiopsis* are parasites of fish–eating birds (Pearson, 2008), so the species cited here probably have a wide distribution range in Argentina.

**Family: Cryptogonimidae Ward, 1917**

*Acanthostomoides apophalliformis* Szidat, 1956

**Hosts and localities:** (metacercariae) †*Jenynsia multidentata –* Neuquén River (Arroyón Stream, downstream Ballester Dam).

(adults) *Oncorhynchus mykiss –* Limay River (Escondido Lake, Moreno Lake, Nahuel Huapi Lake).

*Salmo trutta* – Limay River (Nahuel Huapi Lake, Piedra del Águila Reservoir).

*Salvelinus fontinalis* – Limay River (Moreno Lake, Nahuel Huapi Lake).

**Site of infection:** (metacercariae) liver. (adults) intestinal caeca, intestine.

**Stage:** metacercariae and ingravid adults.

**Origin:** native.

**Other records in Patagonia:** (metacercariae) Ortubay et al*.* (1994) in *Galaxias maculatus* and *Galaxias platei*, Ostrowski de Núñez et al*.* (1999), Revenga and Scheinert (1999), Revenga et al*.* (2005, 2006a, 2006b), Viozzi et al*.* (2009), and Fernández et al*.* (2010, 2015a, 2015b) in *Galaxias maculatus*, and Fernández et al*.* (2012) in *Aplochiton zebra*.

(Adults) Ortubay et al*.* (1994) in *O. mykiss* and *Percichthys trucha*, Ostrowski de Núñez et al*.* (1999) in *O. mykiss*, *Olivaichthys viedmensis* (MacDonagh), and *P. trucha*, Shimazu et al*.* (2000) in *S. trutta*, and *P. trucha*, and Rauque et al*.* (2003) in *O. mykiss*, *S. fontinalis*, *Galaxias platei,* *O. viedmensis*, and *P. trucha*.

**Vouchers:** (metacercariae) UNCo–Pa 86/1–4 (Ostrowski de Núñez et al*.*, 1999), MACN–Pa 456/1–2 (Viozzi et al*.*, 2009), MACN–Pa 524/132 (Fernández et al*.*, 2012). The small amount of worms and/or the state of conservation did not allow the preparation of voucher specimens from the present survey.

(Adults) MACN–Pa 379/1–2, UNCo–Pa 65/1–8, 66 (Ostrowski de Núñéz et al*.*, 1999), NSMT–Pl 4579–4582, IPCAS D–422 (Shimazu et al*.*, 2000), MACN–Pa 641/1 (present survey).

**Remarks:** *A*. *apophalliformis* was originally described from *P. trucha* in Patagonia (Szidat, 1956). Its life cycle includes small fishes as second intermediate hosts and piscivorous fishes as definitive ones. Adults of this parasite species are commonly found in larger fishes that acquired the infection eating the native *G. maculatus*. *Acanthostomoides apophalliformis* is restricted to fishes from Argentinean Patagonia. Salmonids represent a dead end for the life cycle of this parasite, since worms do not reach sexual maturity in these hosts.

**Family: Zoogonidae Odhner, 1902**

*Steganoderma macrophallus* Szidat and Nani, 1951

**Hosts and localities:** †*Odontesthes bonariensis –* Limay River (Gatica Beach).

**Site of infection:** intestine.

**Stage:** gravid adults.

**Origin:** native.

**Other records in Patagonia:** Szidat and Nani (1951), and Flores et al*.* (2016) in *Odontesthes hatcheri*.

**Vouchers:** MLP–He 7139 (Flores et al*.*, 2016), MACN–Pa 640/1 (present survey).

**Remarks:** *S. macrophallus* was originally described from *O. hatcheri* in Limay River (Szidat and Nani, 1951). The record of infected *O. bonariensis* specimens could represent a case of spillback.

*Steganoderma szidati* Viozzi, Flores and Ostrowski de Núñez, 2000

**Hosts and localities:** †*Oncorhynchus mykiss –* Limay River (Moreno Lake).

**Site of infection:** intestine.

**Stage:** gravid adults.

**Origin:** native.

**Other records in Patagonia:** Viozzi et al*.* (2000) in *Galaxias maculatus* and *Galaxias platei*, Revenga et al*.* (2005), Viozzi et al*.* (2009), and Fernández et al*.* (2010, 2015a, 2015b) in *G. maculatus*, and Fernández et al*.* (2012) in *Aplochiton zebra*.

**Vouchers:** MACN–Pa 393/1–3, UNCo–Pa 87; 99/1–19; 100/1–10; 101/1–2; 102/1–3; 103/1–6; 119; 120; 121 (Viozzi et al*.*, 2000), MACN–Pa 457/1–2 (Viozzi et al*.*, 2009), MACN–Pa 527 (Fernández et al*.*, 2012), MACN–Pa 639/1 (present survey).

**Remarks:** *Steganoderma szidati* was originally described in galaxids from Patagonian Andean lakes (Viozzi et al*.*, 2000). This parasite is restricted to fishes from Andean environments of Patagonia, Argentina. The record of specimens infected in Moreno Lake could represent a case of spillback. Ortubay et al*.* (1994) cited an unidentified species of *Steganoderma* parasitizing *O. mykiss* in Lake Rosario, Chubut Province.

**Cestoda**

**Family: Bothriocephalidae Blanchard, 1849**

*Schizocotyle acheilognathi* (Yamaguti, 1934)

**Hosts and localities:** *Cyprinus carpio* – Neuquén River (downstream Ballester Dam), Limay River (downstream Arroyito Dam), Negro River (Allen City, Guardia Mitre Town).

**Site of infection:** intestine.

**Stage:** gravid adults.

**Prevalence (P) and mean intensity (MI):** downstream Ballester Dam (N=55, P=16%, MI=4.4); downstream Arroyito Dam (N=22, P=4.5%, MI=1); Allen City (N=52, P=40%, MI=1.3); Guardia Mitre Town (N=61, P=1.6%, MI=1).

**Origin:** co–introduced with *Cyprinus carpio*.

**Date of first detection in Patagonia:** November 2011.

**Other records in Patagonia:** Waicheim et al*.* (2014) in *C. carpio*.

**Vouchers:** MACN–Pa 561/1–2 (Waicheim et al*.*, 2014), MACN–Pa 636/1 (present survey).

**Remarks:** *S. acheilognathi* has been introduced worldwide along with cyprinids; it is known as the most successful invasive parasite in freshwater (Scholz et al*.*, 2012), and highly pathogenic for native fishes (Velázquez–Velázquez et al*.*, 2011). In Argentina this parasite has only been reported from Neuquén, Limay and Negro Rivers in Patagonia, where dams seems to constitute a barrier to its dispersion upstream. Cited by Waicheim et al*.* (2014) as *Bothriocephalus* sp.

**Family: Diphyllobothriidae Lühe, 1910**

*Dibothriocephalus* sp.

**Syn.:** *Diphyllobothrium* sp*.*

**Hosts and localities:** *Oncorhynchus mykiss –* Limay River (Escondido Lake, Morenito Lake, Moreno Lake, Gutiérrez Lake, Nahuel Huapi Lake, Traful Lake), Futaleufú River (Cholila Lake).

*Salmo salar* – Limay River (Alicura Reservoir).

*Salmo trutta* – Limay River (Moreno Lake, Gutiérrez Lake, Nahuel Huapi Lake, Alicura Reservoir, Piedra del Águila Reservoir).

*Salvelinus fontinalis* – Limay River (Moreno Lake, Gutiérrez Lake, Nahuel Huapi Lake).

**Site of infection:** abdominal cavity.

**Stage:** plerocercoid.

**Prevalence (P) and mean intensity (MI):**

*Oncorhynchus mykiss*: Escondido Lake (N=8, P=63%, MI=9.2); Morenito Lake (N=1, P=1/1, MI=1); Moreno Lake (N=125, P=25%, MI=15.4); Gutiérrez Lake (N=13, P=69%, MI=28.1); Nahuel Huapi Lake (N=131, P=69%, MI=29.6); Traful Lake (N=3, P=2/3, MI=89.5); Cholila Lake (N=7, P=14%, MI=1).

*Salmo salar*: Alicura Reservoir (N=6, P=100%, MI=5.8).

*Salmo trutta*: Moreno Lake (N=3, P=1/3, MI=3); Gutiérrez Lake (N=2, P=2/2, MI=11.5), Nahuel Huapi Lake (N=98, P=72%, MI=9.8); Alicura Reservoir (N=6, P=54%, MI=7.4); Piedra del Águila Reservoir (N=13, P=8%, MI=1).

*Salvelinus fontinalis*: Moreno Lake (N=68, P=16%, MI=1.5); Gutiérrez Lake (N=4, P=3/4, MI=4.3)*.*

**Origin:** introduced.

**Date of first detection in Patagonia:** 1952.

**Other records in Patagonia:** Szidat and Soria (1952) in *S. salar*, *S. fontinalis* and *O. mykiss*, Ortubay et al*.* (1994) in *Oncorhynchus tshawystcha*, *S. trutta* and *P. trucha*, Revenga et al*.* (1995) in *O. mykiss* and *S. fontinalis*, Semenas (2006) in *O. mykiss*, *O. tshawystcha*, *S. fontinalis*, *S. salar*, *S. trutta*, *Galaxias maculatus*, *Galaxias platei*, and *Percichthys trucha*, Fernández et al*.* (2015a, 2015b) in *G. maculatus*.

**Remarks:** Two freshwater species of *Dibothriocephalus* have been recorded in South America, both found in fishes from Patagonia: *D*. *dendriticus* (Revenga and Semenas, 1991; Revenga, 1993; Ortubay et al*.*, 1994; Viozzi et al*.*, 2009) and *D. latus* (Bacigalupo and D’Alessandro Bacigalupo, 1952; Szidat and Soria, 1952, 1957; Szidat, 1964; Revenga and Semenas, 1991; Revenga, 1993). We cited the specimens as *Dibothriocephalus* sp. because it is difficult to differentiate both species based on the morphology of plerocercoids (Andersen and Gibson, 1989; Revenga and Semenas, 1991). Salmonids were introduced more than 100 years ago as embryos, so they arrived without parasites; however, they act as suitable hosts for these cestodes. Adults of *D. dendriticus* have been registered mainly in birds, whereas *D. latus* parasitises mammals, including humans.

*Dibothriocephalus dendriticus* (Nitzsch, 1824)

**Syn:** *Diphyllobothrium dendriticum* Nitzsch, 1824

**Site of infection:** abdominal cavity.

**Stage:** plerocercoid.

**Origin:** introduced.

**Date of first detection in Patagonia:** 1957.

**Other records in Patagonia:** Szidat and Soria (1957) in *O. mykiss*, *S. fontinalis* and *S. salar*, Szidat (1964) in *O. mykiss*, *S. salar*, Revenga and Semenas (1991) in *Oncorhynchus mykiss*, *Salmo trutta*, and *Salvelinus fontinalis*, Revenga (1993) in *O. mykiss* and *S. fontinalis*, Ortubay et al*.* (1994) in *O. mykiss*, *Salmo salar*, and *S. fontinalis*, Viozzi et al*.* (2009) in *Galaxias maculatus*.

**Remarks:** *D. dendriticus* (cited as *Sparganum microcordiceps*) was first recorded in salmonids from Huechulafquen, Moreno, and Nahuel Huapi lakes (Szidat and Soria, 1957). Adults of *D. dendriticus* had been morphologically identified in the seagull (*Larus dominicanus*) and brown–headed gull (*Chroicocephalus maculipennis*) (Szidat, 1964; Kreiter and Semenas, 1997). The specific identity was confirmed later by molecular studies in adults from *L. dominicanus* (Casalins et al., 2015). The origin of this species in South America is unclear, but their original distribution was probably limited to the Northern Hemisphere (Kuchta et al., 2013). This species could be introduced with migratory fish–eating birds.

*Dibothriocephalus latus* (Linnaeus, 1758)

**Syn:** *Diphyllobothrium latum* Linnaeus, 1758

**Site of infection:** abdominal cavity.

**Stage:** plerocercoid.

**Origin:** probably introduced as adults with European immigrants.

**Date of first detection in Patagonia:** 1952.

**Other records in Patagonia:** Bacigalupo and D’Alessandro Bacigalupo (1952), Szidat and Soria (1952), and Szidat (1964) in *Oncorhynchus mykiss*, Szidat and Soria (1957) in *O. mykiss*, *Salmo salar*, and *Salvelinus fontinalis*, Revenga and Semenas (1991) in *O. mykiss*, *Salmo trutta*, and *S. fontinalis*, Revenga (1993) in *O. mykiss* and *S. fontinalis.*

**Remarks:** In Argentina the presence of plerocercoids of *D. latus* (cited as *Diphyllobothrium latum*) was first mentioned in 1952 in introduced *O. mykiss* from Nahuel Huapi Lake (Bacigalupo and D’Alessandro Bacigalupo, 1952). Adults of *D*. *latus* seem to have been introduced by human migratory currents at the end of XIX century and beginning of XX century (Semenas, 2006). This species has been recorded as infecting humans in Southern South America (Semenas, 2006; Torres, 2013).

**Family: Sphyriocephalidae Pintner, 1913**

*#Hepatoxylon* sp.

**Hosts and localities:** *Oncorhynchus tshawytscha* – Corcovado River (Corcovado River).

**Site of infection:** abdominal cavity.

**Stage:** plerocercoid.

**Prevalence (P) and mean intensity (MI):** Corcovado River (N=1, P=1/1, MI=30)*.*

**Origin:** co–introduced with *Oncorhynchus tshawytscha*.

**Date of first detection in Patagonia:** November 1999.

**Remarks:** In South America, 2 species of this genus, *Hepatoxylon megacephalum* (Rudolphi, 1819) Dollfus, 1942 and *Hepatoxylon trichiuri* (Holten, 1802) Bosc, 1811, have been recorded parasitising mainly a wide range of marine fishes, including families of actinopterygians and elasmobranchians (Alves et al., 2017). The larva recorded here is a marine parasite that reaches Andean Patagonian rivers in naturalized populations of introduced chinook salmons that make annual migrations from the Southern Pacific Ocean to the headwaters of rivers located east of the Andes. *Oncorhynchus tshawytscha* has also been cited as host in Southern Chile (Reyes–Piraino, 1982).

**Family: Proteocephalidae La Rue, 1911**

*Cangatiella macdonaghi* (Szidat and Nani, 1951)

**Syn.:** *Ichthyotaenia macdonaghi* Szidat and Nani, 1951, *Proteocephalus macdonaghi* (Szidat and Nani, 1951) Yamaguti, 1959.

**Hosts and localities:** *Odontesthes bonariensis –* Colorado River (Casa de Piedra Reservoir), Neuquén River (Pellegrini Lake).

**Site of infection:** intestine.

**Stage:** gravid adults.

**Origin:** native.

**Other records in Patagonia:** Szidat and Nani (1951), Ortubay et al*.* (1994), Gil de Pertierra and Viozzi (1999), and Flores et al*.* (2016) in *Odontesthes hatcheri*.

**Vouchers:** MACN–Pa 378/1–5, MHNG INVE 22842–4, UNCo–Pa 72–73 (Gil de Pertierra and Viozzi 1999), MLP–He7135 (Flores et al*.* 2016), MACN–Pa 635/1 (present survey).

**Remarks:** Szidat and Nani (1951) described this cestode from the native *O. hatcheri* (citing this host species as *Basilichthys microlepidotus*) in Pellegrini Lake; Gil de Pertierra and Viozzi (1999) later redescribed the species from the same host and locality. This species was recorded in *O. bonariensis* from Córdoba (Bethular et al*.*, 2014), and Buenos Aires Provinces (Flores et al*.*, 2016). This parasite species has been recorded in *O. bonariensis* from Central Argentina and from Northern Patagonia, and in the Patagonian *O. hatcheri*; molecular studies are required to verify if they belong to the same species.

**Family: Triaenophoridae Lönnberg, 1899**

*Ailinella mirabilis* Gil de Pertierra and Semenas, 2006

**Hosts and localities:** *Oncorhynchus mykiss –* Limay River (Escondido Lake, Moreno Lake, Nahuel Huapi Lake).

*Salvelinus fontinalis* – Limay River (Moreno Lake).

**Site of infection:** intestine.

**Stage:** non–gravid adults.

**Origin:** native.

**Other records in Patagonia:** Ortubay et al*.* (1994) in *Aplochiton zebra* and *Galaxias maculatus*, Revenga et al*.* (2005), Gil de Pertierra and Semenas (2006), Viozzi et al*.* (2009), Fernández et al*.* (2010, 2015a, 2015b) in *G. maculatus*, Rauque et al*.* (2003) in *O. mykiss*, *S. fontinalis*, *G. maculatus*, *Galaxias platei*, and *Percichthys trucha*, and Fernández et al*.* (2012) in *A. zebra*.

**Vouchers:** MACN–Pa 434/1–7, IPCAS C–438 (Gil de Pertierra and Semenas 2006), MACN–Pa 528/1–2 Fernández et al*.* (2012), the small amount of worms and/or the state of conservation did not allow the preparation of voucher specimens from the present survey.

**Remarks:** *Ailinella mirabilis* is generally found in native galaxiids from Patagonia. Salmonids could represent a sink in the life cycle of this parasite. Cited as *Nippotaenia* sp. by: Ortubay et al*.* (1994), Rauque et al*.* (2003), and Revenga et al*.* (2005). Host cited by Ortubay et al*.* (1994) as *A. taeniatus*. Erroneously cited as larvae by Fernández et al*.* (2015b).

**Nematoda**

**Family: Anisakidae Railliet and Henry, 1912**

*Contracaecum* sp.

**Hosts and localities:** *Oncorhynchus mykiss* – Neuquén River (El Chañar Reservoir, downstream Ballester Dam), Limay River (Escondido Lake, Moreno Lake, Nahuel Huapi Lake, Traful Lake, Alicura Reservoir, Piedra de Águila Reservoir, Ramos Mexía Reservoir, downstream Arroyito Dam), Futaleufú River (Cholila Lake).

†*Salmo salar* – Limay River (Piedra del Águila Reservoir).

*Salmo trutta* – Limay River (Moreno Lake, Nahuel Huapi Lake, Piedra del Águila Reservoir, Pichi Picún Leufú Reservoir).

†*Salvelinus fontinalis* – Limay River (Moreno Lake).

*Cyprinus carpio* – Neuquén River (downstream Ballester Dam), Limay River (downstream Arroyito Dam, Herradura Backwater, Negro River (Allen City, Guardia Mitre Town).

†*Cheirodon interruptus* – Neuquén River (Arroyón Stream), Valcheta Stream (Chipauquil Town).

†*Cnesterodon decemmaculatus –* Valcheta Stream (Chipauquil Town).

†*Jenynsia multidentata –* Colorado River (Casa de Piedra Reservoir), Neuquén River (Arroyón Stream, dowstream Ballester Dam).

*Odontesthes bonariensis –* Colorado River (Casa de Piedra Reservoir), Neuquén River (Pellegrini Lake).

**Site of infection:** abdominal cavity.

**Stage:** larva.

**Origin:** native.

**Other records in Patagonia:** Szidat and Nani (1951) in *Odontesthes hatcheri*, Ortubay et al*.* (1994) in *O. mykiss*, *Galaxias maculatus*, *Galaxias platei*, *Olivaichthys viedmensis*, and *O. hatcheri*, Revenga and Scheinert (1999), Revenga et al*.* (2005), Viozzi et al*.* (2009)*,* and Fernández et al*.* (2010, 2015a, 2015b) in *G. maculatus,* Fernández et al*.* (2012) in *Aplochiton zebra*, Waicheim et al*.* (2014) in *C. carpio*, and Flores et al*.* (2016) in *O. hatcheri*.

**Vouchers:** MACN–Pa 464 (Viozzi et al*.*, 2009), MACN–Pa 529 (Fernández et al*.*, 2012), MACN–Pa 562/1 (Waicheim et al*.*, 2014).

**Remarks:** We provide the first record of this common larva in *S. salar*, *S. fontinalis*, *C. interruptus*, *C. decemmaculatus*, and *J. multidentata* from South America. Species of the genus *Contracaecum* have been cited in many freshwater fishes from Central and Northern Argentina (Hamman, 1999; Ramallo and Torres, 1995; Mancini et al*.*, 2000, 2005, 2006, 2008, 2009, 2014; García Romero, 2001; Tanzola et al*.*, 2009; Drago, 2012; and Flores et al*.*, 2016). *Contracaecum* are parasites of fish–eating birds and marine mammals (Moravec, 1998), so the species of this genus probably have a wide distribution range in Argentina.

*Goezia* sp.

**Site of infection:** abdominal cavity.

**Stage:** larva?

**Origin:** native.

**Other records in Patagonia:** Ortubay et al*.* (1994) in *Oncorhychus mykiss* and *Odontesthes bonariensis*.

**Remarks:** Ortubay et al*.* (1994) did not indicate the stage of development of this nematode, although considering site of infection it can be inferred that it is a larval form in a paratenic host. In Argentina species of the genus *Goezia* have been cited as parasitizing the gut of freshwater fishes from the Paraná River (Moravec, 1998). It is possible that this species has a wide distribution range, from Parana River to the south, reaching the Colorado River, the northernmost limit of Patagonia.

*Hysterothylacium patagonense* Moravec, Urawa and Coria, 1997

**Hosts and localities:** *Oncorhynchus mykiss –* Hua–Hum River (Lacar Lake), Limay River (Escondido Lake, Moreno Lake, Nahuel Huapi Lake), Grande River (Grande River).

*Salmo trutta* – Limay River (Moreno Lake, Nahuel Huapi Lake).

*Salvelinus fontinalis* – Limay River (Moreno Lake, Nahuel Huapi Lake).

**Site of infection:** intestinal caeca, intestine.

**Stage:** non gravid adults.

**Origin:** native.

**Other records in Patagonia:** Ortubay et al*.* (1994) in *Odontesthes hatcheri* and *Percichthys trucha*, Moravec et al*.* (1997) in *O. mykiss*, *S. trutta*, *S. fontinalis*, and *P. trucha*, Rauque et al*.* (2003) in *O. mykiss*, *S. fontinalis*, *Galaxias platei*, *Olivaichthys viedmensis*, and *P. trucha*, Viozzi et al*.* (2009) and Fernández et al*.* (2010, 2015a, 2015b) in *Galaxias* *maculatus*, Fernández et al*.* (2012) in *Aplochiton zebra*, and Flores et al*.* (2016) in *O. hatcheri*.

**Vouchers:** IPCAS 675, BMNH 1996.5.30–1–5, MPM 19695 (Moravec et al*.*, 1997), MACN–Pa 465(Viozzi et al*.*, 2009), MACN–Pa 655/1 (present survey).

**Remarks:** *Hysterothylacium patagonense* use the native *G. maculatus* as second intermediate host (Viozzi et al., 2009), and the native *P. trucha* as definitive host (Moravec et al*.*, 1997). This parasite occurred only in Patagonian fishes. Cited as *Hysterothylacium* sp. by Ortubay et al*.* (1994). Parasitised salmonids represent a sink for the parasite life cycle.

**Family: Hedruridae Nitzsch, 1812**

*Hedruris suttonae* Brugni and Viozzi, 2010

**Hosts and localities:** *Oncorhynchus mykiss –* Limay River (Moreno Lake, Nahuel Huapi Lake), Senguer River (Musters Lake).

*Salmo trutta* – Limay River (Nahuel Huapi Lake).

†*Salvelinus fontinalis* – Limay River (Moreno Lake, Nahuel Huapi Lake), Futaleufú River (Cholila Lake).

**Site of infection:** stomach.

**Stage:** gravid adults.

**Origin:** native

**Other records in Patagonia**: Ortubay et al*.* (1994) in *O. mykiss*, *S. trutta*, *Galaxias* *platei*, and *Percichthys trucha*, Revenga et al*.* (2005), Viozzi et al*.* (2009), and Fernández et al*.* (2010, 2015a, 2015b) in *Galaxias maculatus*, and Brugni and Viozzi (2010) in *G. maculatus* and *G. platei.*

**Vouchers:** MACN–Pa 466 (Viozzi et al*.*, 2009), MACN–Pa 406/1–2, UNCo–Pa 129, MLP–He 4927, USNPC 102470 (Brugni and Viozzi, 2010), MACN–Pa 656/1 (present survey).

**Remarks:** adults of *H. suttonae* were originally described in the galaxiids *G. maculatus* and *G. platei* from Patagonia (Brugni and Viozzi, 2010). The intermediate host is the amphipod *Hyalella patagonica.* Its presence in salmonids probably represent a case of spillback. This parasite is restricted to fishes from Argentinean Patagonia. Cited as *Hedruris* sp. by Ortubay et al*.* (1994), Revenga et al*.* (2005), and Viozzi et al*.* (2009).

**Family: Camallanidae Railliet and Henry, 1915**

*Camallanus corderoi* Torres, Teuber and Miranda, 1990

**Hosts and localities:** *Oncorhynchus mykiss –* Neuquén River (El Chañar Reservoir), Limay River (Escondido Lake, Morenito Lake, Moreno Lake, Nahuel Lake, Ramos Mexía Reservoir, downstream Arroyito Dam).

†*Salmo salar* – Limay River (Alicura Reservoir).

†*Salmo trutta* – Limay River (Moreno Lake, Gutiérrez Lake, Nahuel Huapi Lake, Alicura Reservoir, Piedra del Águila Reservoir, Pichi Picún Leufú Reservoir).

*Salvelinus fontinalis* – Limay River (Moreno Lake).

†*Jenynsia multidentata –* Neuquén River (Arroyón Stream).

†*Odontesthes bonariensis* – Neuquén River (Pellegrini Lake).

**Site of infection:** intestine.

**Stage:** gravid adults in *Odontesthes bonariensis* and non gravid adults in the other fish species.

**Origin:** native.

**Other records in Patagonia:** Szidat (1956) in *P. trucha*, Ortubay et al*.* (1994) in *O. mykiss*, *S. fontinalis*, *Galaxias maculatus*, *Odontesthes hatcheri*, and *Percichthys trucha*, Revenga and Scheinert (1999) in *G. maculatus*, Rauque et al*.* (2003) in *O. mykiss*, *S. fontinalis*, *G. maculatus*, *Galaxias platei*, and *P. trucha*, Viozzi et al*.* (2009) and Fernández et al*.* (2010, 2015a, 2015b) in *G. maculatus*, Fernández et al*.* (2012) in *Aplochiton zebra*, and Flores et al*.* (2016) in *O. hatcheri*.

**Vouchers:** MACN–Pa 463 (Viozzi et al*.*, 2009), MACN–Pa 531 Fernández et al*.* (2012), MACN–Pa 657/1 (present survey).

**Remarks:** in Argentinean Patagonia, *C. corderoi* uses the native *G. maculatus* as intermediate host and the native *P. trucha* as definitive (Torres et al*.*, 1990; Rauque et al*.*, 2003; Viozzi et al*.*, 2009; Fernández et al*.*, 2010, 2015a, 2015b). Salmonids seem to represent a sink for the parasite. *Camallanus corderoi* is distributed in freshwater fishes from the South of Chile and Argentina (Moravec, 1998). Cited as *Camallanus tridentatus* by Szidat (1956).

**Family: Guyanemidae Petter, 1974**

*Pseudodelphis limnicola* Brugni and Viozzi, 2006

**Hosts and localities:** †*Oncorhynchus mykiss –* Limay River (Ramos Mexía Reservoir).

**Site of infection:** sinus venosus.

**Stage:** ingravid adult.

**Origin:** native.

**Other records in Patagonia:** Brugni and Viozzi (2006) in *Percichthys trucha*.

**Vouchers:** MACN–Pa 429/1–10, MLP–He 5519, UNCo–Pa 201/1–20, IPCAS N–833 (Brugni and Viozzi, 2006), the small amount of worms and/or the state of conservation did not allow the preparation of voucher specimens from the present survey.

**Remarks:** Moravec et al*.* (1997) described the philometrid *Philonema percichthydis,* taken from the abdominal cavity of *P. trucha* in Patagonia, based only on the morphology of two females. It would be useful to know the morphology of the males of this species, since it could be conspecific with the guyanemid found in the present work and previously described by Brugni and Viozzi (2006). *Pseudodelphis limnicola* is restricted to fishes from Argentinean Patagonia. Infected *O. mykiss* probably represent a sink for the parasite life cycle.

**Family: Rhabdochonidae Skrjabin, 1946**

*Rhabdochona* sp.

**Site of infection:** intestine.

**Stage:** adult.

**Origin:** native.

**Other records in Patagonia:** Ortubay et al*.* (1994) in *Oncorhynchus mykiss*.

**Remarks:** This species probably corresponds to *Rhabdochona* *acuminata,* which was reported from *Olivaichthys* *mesembrinus* in Chubut River (Cremonte et al., 2002).

**Acanthocephala**

**Family: Echinorhynchidae Cobbold, 1879**

*Acanthocephalus tumescens* (von Linstow, 1896)

**Syn.:** *Echinorhynchus tumescens* von Linstow, 1896.

**Hosts and localities:** *Oncorhynchus mykiss –* Limay River (Escondido Lake, Moreno Lake, Gutiérrez Lake, Nahuel Huapi Lake), Puelo River (Epuyén Lake), Futaleufú River (Cholila Lake, Amutui Quimey Reservoir), Corcovado River (Corcovado River).

*Salmo trutta* – Limay River (Moreno Lake, Nahuel Huapi Lake), Puelo River (Epuyén Lake).

*Salvelinus fontinalis* – Limay River (Moreno Lake, Nahuel Huapi Lake), Futaleufú River (Cholila Lake).

**Site of infection:** intestine.

**Stage:** gravid adults.

**Origin:** native.

**Other records in Patagonia:** von Linstow (1896) in *Odontesthes* sp., Szidat and Soria (1957) in *O. mykiss*, Ortubay et al*.* (1994) in *O. mykiss*, *Salmo salar*, *S. trutta*, *S. fontinalis*, *Salvelinus namaycush*, *Galaxias maculatus*, *Galaxias platei*, *Olivaichthys viedmensis*, *Odontesthes hatcheri*, and *Percichthys trucha*, Semenas and Trejo (1997), Trejo et al*.* (2000), Rauque et al*.* (2002), Revenga et al*.* (2005), Viozzi et al*.* (2009), Paterson et al*.* (2013), and Fernández et al*.* (2015a, 2015b) in *G. maculatus*, Rauque et al*.* (2003) in *O. mykiss*, *S. trutta*, *S. fontinalis*, *G. maculatus*, *G. platei*, *O. hatcheri*, and *P. trucha*, Rauque et al*.* (2006) in *O. mykiss*, *G. platei*, and *P. trucha*, Fernández et al*.* (2012) in *Aplochiton zebra*, and Flores et al*.* (2016) in *O. hatcheri*.

**Vouchers:** MACN–Pa 386 (Semenas and Trejo, 1997), MACN–Pa 461/1–2 (Viozzi et al*.*, 2009), MACN–Pa 530/1–2, UNCo–Pa 81–84 (Fernández et al*.*, 2012), MACN–Pa 634/6 (present survey).

**Remarks:** Cited by von Linstow (1896) as *Echinorhynchus tumescens* in *Odontesthes hatcheri* (Semenas and Trejo, 1997). Cited as *Echinorhynchus* spp. by Szidat and Soria (1957). This parasite has low host specificity and is restricted to freshwater environments of Chilean and Argentinean Patagonia (Torres et al., 1992; Viozzi et al., 2009). Their presence in salmonids is a case of spillback (Rauque et al*.*, 2003).

**Family: Polymorphidae Meyer, 1931**

*Polymorphus* sp. 1

**Hosts and localities:** #*Jenynsia multidentata –* Neuquén River (Arroyón Stream, downstream Ballester Dam).

**Site of infection:** intestinal wall.

**Stage:** cystacanth.

**Origin:** unknown.

**Remarks:** The species found in this study resembles *Polymorphus inermis* (Travassos, 1923) in that it has a low number of hooks (4 per row) in the foretrunk, but differs from it by having 12 hooks per row in the proboscis (6 in *P. inermis*). This parasite is probably a new species. The only previous record of a cystacanth of *Polymorphus* sp. in Argentinean fishes correspond to Waicheim et al*.* (2014) parasitizing *C. carpio*, but the larva reported here differs from those of common carp by having lower numbers of hooks per row in the proboscis and in the foretrunk (see *Polymorphus* sp. 2).

*Polymorphus* sp. 2

**Hosts and localities:** *Oncorhynchus mykiss* – Futaleufú River (Cholila Lake).

*Cyprinus carpio* – Neuquén River (downstream Ballester Dam), Limay River (Allen).

*Cheirodon interruptus* – Neuquén River (Arroyón).

*Corydoras paleatus –* Neuquén River (Arroyón).

**Site of infection:** liver, intestinal wall, abdominal cavity.

**Stage:** cystacanth.

**Origin:** unknown.

**Other records in Patagonia:** Waicheim et al*.* (2014) in *C. carpio*.

**Vouchers**: MACN–Pa 563/1 (Waicheim et al*.*, 2014).

**Remarks:** This species resembles *Polymorphus mutabilis* (Travassos, 1926) or *Polymorphus brevis* (van Cleave, 1916), recorded in fishes from México (Salgado–Maldonado, 1980), as it has 16 hooks per row in the proboscis and 12 hooks per row in the foretrunk. It is unlikely that the range of the North American species reaches the south of South America, so molecular studies are necessary to determine whether it is a new species. The species of *Polymorphus* are parasites of birds and mammals (Amin, 1992), so probably have a wide distribution in Argentina, and the origin of these larvae in Patagonian fishes cannot be determined.

**Family: Pomphorhynchidae Yamaguti, 1939**

*Pomphorhynchus patagonicus* Ortubay, Semenas, Úbeda and Kennedy, 1991

**Hosts and localities:** *Oncorhynchus mykiss* – Limay River (Piedra del Águila Reservoir, Ramos Mexía Reservoir, downstream Arroyito Dam), Futaleufú River (Cholila Lake, Amutui Quimey Reservoir), Senguer River (Musters Lake).

*Salmo trutta* – Limay River (Pichi Picún Leufú Reservoir).

*Salvelinus fontinalis* – Futaleufú River (Cholila Lake).

*Cyprinus carpio* – Neuquén River (downstream Ballester Dam), Limay River (downstream Arroyito Dam, Plottier City), Negro River (Allen City).

**Site of infection:** intestine in salmonids, liver and intestine in *C. carpio*.

**Stage:** immature adults.

**Origin:** native.

**Other record in Patagonia:** Ortubay et al*.* (1991) in *O. mykiss*, *S. fontinalis*, *Galaxias platei*, *Odontesthes hatcheri*, and *Percichthys trucha*, Semenas et al*.* (1992) in *O. mykiss*, *G. platei*, and *O. hatcheri*, Trejo (1992) in *O. mykiss* and *O. hatcheri*, Ortubay et al*.* (1994) in *O. mykiss*, *S. fontinalis*, *A. zebra*, *G. platei*, *O. viedmensis*, *O. hatcheri*, and *P. trucha*, Úbeda et al*.* (1994) in *O. mykiss*, *G. platei*, and *O. hatcheri*, Viozzi et al*.* (2009) and Fernández et al*.* (2015a) in *Galaxias maculatus*, Fernández et al*.* (2012) in *A. zebra*, Waicheim et al*.* (2014) in *C. carpio*, and Flores et al*.* (2016) in *O. hatcheri*.

**Vourchers:** MACN–Pa 358 (Ortubay et al*.*, 1991), MACN–Pa 462 (Viozzi et al*.*, 2009), MACN–Pa 564/1 (Waicheim et al*.*, 2014), MACN–Pa 633/3 (present survey).

**Remarks:** This parasite is distributed in freshwater environments of Argentinean Patagonia, and matures in native fishes, especially *O. hatcheri*, *G. platei* and *P. trucha* (Trejo, 1994; Úbeda et al*.*, 1994). Alien fishes represent a sink for the parasite life cycle (Trejo, 1994; Úbeda et al*.*, 1994; Waicheim et al*.*, 2014).

**References**

Acosta, A.A., Queiroz, J., Brandão, H., Carvalho, E., da Silva, R.J. 2013. Helminths of *Steindachnerina insculpta* in two distinct stretches of the Taquari River, state of São Paulo, Brazil. Rev. Bras. Parasitol. V. 22, 539–547.

Acosta, A.A., Queiroz, J., Brandão, H., da Silva, R.J. 2015. Helminth fauna of Astyanax fasciatus Cuvier, 1819, in two distinct sites of the Taquari River, São Paulo State, Brazil. [Braz. J. Biol](http://www.scielo.br/scielo.php?script=sci_serial&pid=1519-6984&lng=en&nrm=iso). 75, 242–250.

Alves, P.V., de Chambrier, A., Scholz, T., Luque, J.L. 2017. Annotated checklist of fish cestodes from South America. ZooKeys 650, 1–205.

Amin, O.M. 1992. Review of the genus *Polymorphus* Luhe, 1911 (Acanthocephala: Polymorphidae), with the synonymization of *Hexaglandula* Petrochenko, 1950, and *Subcorynosoma* Hoklova, 1967, and a Key to the species. Qatar University Science Journal 12, 115–123.

Andersen, K.I., Gibson, D.I. 1989. A key of three species of larval *Diphyllobothrium* Cobbold, 1858 (Cestoda: Pseudophyllidea) occurring in European and North American freshwater fishes. Syst. Parasitol. 13, 3–9.

Bacigalupo, J., D'alessandro Bacigalupo, A. 1952. Difilobotriasis autóctona del perro en la Argentina. Gaceta Veterinaria 14, 216–222.

Bethular, A., Mancini, M., Salinas, V., Echaniz, S., Vignatti, A., Larriestra, A. 2014. Alimentación, condición corporal y principales parásitos del pejerrey (*Odontesthes bonariensis*) del embalse San Roque (Argentina). Biología Acuática 30, 59–68.

Blasco–Costa, I., Poulin, R., Presswell, B. 2017. Morphological description and molecular analyses of *Tylodelphys* sp. (Trematoda: Diplostomidae) newly recorded from the freshwater fish *Gobiomorphus cotidianus* (common bully) in New Zealand. J. Helminthol. 91, 332–345.

Brugni, N., Viozzi, G. 2006. *Pseudodelphis limnicola* sp. n. (Dracunculoidea: Guyanemidae) from the heart of *Percichthys trucha* (Perciformes: Percichthyidae) in Patagonian Andean lakes (Argentina). Folia Parasitol. 53, 134–138.

Brugni, N., Viozzi, G. 2010. A new hedrurid species (Nematoda) from galaxiid fishes in Patagonia (Argentina) and infection of amphipods as intermediate host. J. Parasitol. 96, 109–115.

Bueno–Silva, M., Boeger, W.A. 2009. Neotropical Monogenoidea. 53. *Gyrodactylus corydori* sp. n. and redescription of *Gyrodactylus anisopharynx* (Gyrodactylidea: Gyrodactylidae), parasites of *Corydoras* spp. (Siluriformes: Callichthyidae) from southern Brazil. Folia Parasitol. 56, 13–20.

Casalins, L., Arbetman, M., Semenas, L., Veleizán, A., Flores, V., Viozzi, G. 2015. Difilobotriosis en gaviotas. Pasado y presente de esta zoonosis en el Parque Nacional Nahuel Huapi. Revista Argentina de Zoonosis y Enfermedades Infecciosas Emergentes 10, 38–39.

Cremonte, F., Navone, G.T., Gosztonyi, A.E., Kuba, L. 2002. Redescription of *Rhabdochona* (*Rhabdochona*) *acuminata* (Nematoda: Rhabdochonidae) from freshwater fishes from Patagonia (Argentina), the geographical implications. J. Parasitol. 88, 934–941.

Cussac, V.E., Fernández, D.A., Gómez, S.E., López, H.L. 2009. Fishes of southern South America: a story driven by temperature. Fish Physiol. Biochem. 35, 29–42.

Da Silva Silveira De Almeida, K., Cohen, S. 2011. Diversidade de Monogenea (Platyhelminthes) parasitos de *Astyanax altiparanae* do reservatório da usina hidrelétrica de Itaipu. Saúde, Ambiente 6, 31–41.

Doma, I.L., Ostrowski de Nuñez, M. 1994. Biología poblacional de *Posthodiplostomum nanum* Dubois, 1937 (Trematoda, Diplostomidae) en *Jenynsia lineata* y *Cnesterodon decemmaculatus* (Pisces, Atheriniformes), de la Laguna Chis–Chis, Provincia de Buenos Aires, Argentina*.* Rev. Bras. Biol. 54, 669–679.

Drago, F.B. 2012. Community structure of metazoan parasites of silverside, *Odontesthes bonariensis* (Pisces, Atherinopsidae) from Argentina. Iheringia, Ser. Zool. 102, 26–32.

Drago, F.B., Lunaschi, L.I. 2011. Digenean parasites of Ciconiiform birds from Argentina. Rev. Mex. Biodivers. 82, 77–83.

Fayton, T.J., Kritsky, D.C. 2013. *Acolpenteron willifordensis* n. sp. (Monogenoidea: Dactylogyridae) parasitic in the kidney and ureters of the spotted sucker *Minytrema melanops* (Rafinesque) (Cypriniformes: Catostomidae) from Econfina Creek, Florida. Comp. Parasitol. 80, 1–8.

Fernández, M.V., Brugni, N.L., Viozzi, G.P., Semenas, L. 2010 The relationship between fish assemblages and the helminthes communities of a prey fish, in a group of small shallow lakes. J. Parasitol. 96, 1066–1071.

Fernández, V., Semenas, L., Viozzi, G. 2012. Parasites of the “Peladilla,” *Aplochiton zebra* (Osmeriformes: Galaxiidae), from Patagonia (Argentina and Chile). Comp. Parasitol. 79, 231–237.

Fernández, V., Garibotti, G., Semenas, L., Viozzi, G. 2015a. Influence of biotic and abiotic factors on the metazoan parasite communities of a native prey fish: study in 28 Andean Patagonian lakes. Ecología Austral 25, 221–230.

Fernández, V., Semenas, L., Viozzi, G. 2015b. La estructura de las comunidades de helmintos de *Galaxias maculatus* (Osmeriformes: Galaxiidae) en diferentes sitios de un lago de la Patagonia Argentina. Ecología Austral 25, 212–220.

Flores, V. 1997. Aportes a la descripción morfológica de *Tylodelphys barilochensis* y *Tylodelphys* *crubensis* (Trematoda, Diplostomidae) parásitos del encéfalo de *Galaxias* *maculatus* (Teleostei, Galaxiidae). Boletín Chileno de Parasitología 52, 84–88.

Flores, V., Baccalá, N. 1998. Multivariate analysis in the taxonomy of two *Tylodelphys* species Diesing, 1850 (Trematoda, Diplostomidae) from *Galaxias* *maculatus* (Teleostei, Galaxiidae). Syst. Parasitol. 40, 221–227.

Flores, V., Semenas, L. 2002. Infection patterns of *Tylodelphys* *barilochensis* and *T.* *crubensis* (Trematoda, Diplostomatidae) metacercariae in *Galaxias* *maculatus* (Osmeriformes: Galaxiidae) from two Patagonian lakes and observations on their geographical distribution in the Southern Andean Patagonian Region, Argentina. J. Parasitol. 88, 1135–1139.

Flores V., Brugni, N., Ostrowski de Núñez, M. 2004. *Allocreadium pichi* n. sp. (Trematoda: Allocreadiidae) in *Galaxias maculatus* (Osteichthyes: Galaxiidae) from lake Moreno in Patagonia (Argentina). Syst. Parasitol. 58, 217–221.

Flores, V., Semenas, L., Rauque, C, Vega, R., Fernández, V., Lattuca, M. 2016. Macroparasites of silversides (Atherinopsidae: *Odontesthes*) in Argentina. Revista Mexicana de Biodiversidad, 87, 919–927.

Fuster de Plaza, M.L., Boschi, E.E. 1957. Desnutrición y deformaciones vertebrales en pejerreyes de los embalses de Córdoba. Ministerio de Agricultura y Ganadería, Departamento de Investigaciones Pesqueras, Buenos Aires.

García Romero, N. 2001. Alteraciones patológicas del pejerrey (*Odontesthes bonariensis*) en ambientes naturales y bajo condiciones de cultivo. In: Grosman, F. (Ed.), Fundamentos biológicos, económicos y sociales para una correcta gestión del recurso pejerrey. Editorial Astyanax, Azul, pp. 80–90.

Gibson, D.I. 2002. Family Derogenidae. In: Gibson, D., Jones, A., Bray, R.A. (Eds.), Keys to the Trematoda (Vol. 1). CABI Publishing, London, pp. 351–368.

Gibson, D.I., Jones, A., Bray, R.A. 2002. Keys to the Trematoda. Vol. 1. CABI Publishing, Wallingford.

Gil de Pertierra, A.A., Semenas, L.G. 2006. *Ailinella mirabilis* gen. n., sp. n. (Eucestoda: Pseudophyllidea) from *Galaxias maculatus* (Pisces: Galaxiidae) in the Andean–Patagonian region of Argentina. [Folia Parasitol.](https://www.ncbi.nlm.nih.gov/pubmed/17256203) 53, 276–86.

Gil de Pertierra, A.A., Viozzi, G. 1999. Redescription of *Cangatiella macdonaghi* (Szidat and Nani, 1951) comb. nov. (Cestoda: Proteocephalidae) a parasite of the Atheriniform fish *Odontesthes hatcheri* (Eigenmann, 1909) from the Patagonian region of Argentina. Neotrópica 45, 13–20.

Hamman, M.I. 1999. Ecological aspects between *Contracaecum* sp. (Nematoda, Anisakidae) and the host *Serrasalmus spilopleura* Kner, 1860 (Pisces, Characidae) in natural populations of Northeastern Argentina. *Bol. Chil. Parasitol.* 54, 74–82.

Hoffman, G.L. 1999. Parasites of North American freshwater fishes. Cornell University Press, Ithaca.

Holcman–Spector, B., Olagüe, G. 1989. Digenetic trematodes of the genus *Stephanoprora* Odhner, 1902 of birds of Uruguay with the description of two new species. *Acta Parasitol.* 34, 311–317.

Jogunoori, W., Kritsky, D.C., Venkatanarasaiah, J. 2004. Neotropical Monogenoidea. 46. Three new species from the gills of introduced aquarium fishes in India, the proposal of *Heterotylus* n. g. and *Diaphorocleidus* n. g., and the reassignment of some previously described species of *Urocleidoides* Mizelle, Price, 1964 (Polyonchoinea: Dactylogyridae). *Syst. Parasitol.* 58, 115–124.

Kreiter, A., Semenas, L. 1997. Helmintos parásitos de *Larus dominicanus* en la Patagonia Argentina. *Bol. Chil. Parasitol.* 52, 39–42.

Kuchta, R., Brabec, J., Kubáčková, P., Scholz, T. 2013. Tapeworm *Diphyllobothrium dendriticum* (Cestoda) – neglected or emerging human parasite? PLoS Neglect. Trop. D. 7, e2535.

Lunaschi, L.I., Drago, F.B. 2000. *Thometrema patagonica* (Szidat, 1956) n. comb. para *Derogenes patagonicus* (Szidat) Yamaguti, 1971 (Trematoda, Derogenidae). Physis 58, 43–46.

Lunaschi, L.I., Cremonte, F., Drago, F.B. 2007. Checklist of digenean parasites of birds from Argentina. Zootaxa 1403, 1–36.

Mancini, M., Larriestra, A., Sanchez, J. 2000. Estudio ictiopatológico en poblaciones silvestres de la región centro–sur de la provincia de Córdoba, Argentina. Rev.Med. Vet. 81, 104–108.

Mancini, M., Nicola, I., Larriestra, A., Salinas, V., Bucco, C. 2005. Patrones de riesgo e implicancias de la presencia de *Contracaecum* sp. (Nematoda, Anisakidae) en pejerrey *Odontesthes* *bonariensis* (Pisces, Atherinopsidae). Biología Acuática 22, 197–202.

Mancini, M., Rodriguez, C., Prosperi, C., Salinas, V., Bucco, C. 2006. Main diseases of pejerrey (*Odontesthes bonariensis*) in central Argentina. Pesquisa Vet. Brasil. 26, 205–210.

Mancini, M., Bucco, C., Salinas, V., Larriestra, A., Tanzola, R., Guagliardo, S. 2008. Seasonal variation of parasitism in pejerey *Odontesthes bonariensis* (Atheriniformes, Atherinopsidae) from La Viña Reservoir (Córdoba, Argentina). Rev.Bras. Parasitol. V. 17, 28–32.

http://dx.doi.org/10.1590/S1984–29612008000100006

Mancini, M., Nicola, I., Salinas, V., Bucco, C. 2009. Biología del pejerrey *Odontesthes bonariensis* (Pisces, Atherinopsidae) de la laguna Los Charos (Córdoba, Argentina). Rev. Peru. Biol. 15, 65–71.

Mancini, M., Biolé, F., Salinas, V. Guagliardo, S., Tanzola, D., Morra, G. 2014. Prevalencia, intensidad y aspectos ecológicos de *Contracaecum* sp. (Nematode: Anisakidae) en peces de agua dulce de Argentina. Neotrop. Helminthol. 8, 111–122.

Mendoza–Franco, E.F., Aguirre–Macedo, M., Vidal–Martínez, V. 2007. New and previously described species of Dactylogyridae (Monogenoidea) from the gills of Panamanian freshwater fishes (Teleostei). J. Parasitol. 93, 761–771.

Mendoza–Franco, E.F., Reina, R.G., Torchin, M.E. 2009. Dactylogyrids (Monogenoidea) parasitizing the gills of *Astyanax* spp. (Characidae) from Panama and Southeast Mexico, a new species of *Diaphorocleidus* and a proposal for *Characithecium* n. gen. J. Parasitol. 95, 46–55.

Molnár, K. 2012. Fifty years of observations about the changes of *Dactylogyrus* infection of European common carp (*Cyprinus carpio carpio* L.) in Hungary. Magy. Allatorvosok 134, 111–118.

Moravec, F. 1998. Nematodes of freshwater fishes of the Neotropical Region. Academia, Prague.

Moravec, F., Urawa, S., Coria, C.O. 1997. *Hysterothylacium patagonense* n. sp. (Nematoda: Anisakidae) from freshwater fishes in Patagonia, Argentina, with a key to the species of *Hysterothylacium* in American freshwater fishes. [Syst. Parasitol*.*](https://link.springer.com/journal/11230) 36, 31–38.

Moreira, J., Scholz, T., Luque, J.L. 2016. A new species of *Diaphorocleidus* (Monogenea: Ancyrocephalinae) from the gills of *Argonectes robertsi* (Characiformes) and new records of dactylogyrids parasitic on fishes from the Xingu River, Amazon Basin, Brazil. Zoologia-Curitiba 33, e20160022.

Ortubay, S., Úbeda, C., Semenas, L., Kennedy, C. 1991. *Pomphorhynchus patagonicus* n. sp. (Acanthocephala: Pomphorhynchidae) from freshwater fishes of Patagonia, Argentina. J. Parasitol. 77, 353–356.

Ortubay, S., Semenas, L., Úbeda, C. 1989. A study of helminth parasites and their effect on fishes from Rosario Lake (Chubut, Argentina). Riv.Ital. Acquac. 24, 207–218.

Ortubay, S.G., Semenas, L.G., Úbeda, C.A., Quaggiotto, A.E., Viozzi, G.P. 1994. Catálogo de peces dulceacuícolas de la Patagonia argentina y sus parásitos metazoos. Dirección de Pesca de Río Negro, Viedma.

Ostrowski de Núñez, M. 1968. Sobre el ciclo biológico de *Austrodiplostomum mordax* Szidat–Nani 1951. Neotrópica 14, 85–88.

Ostrowski de Núñez, M. 1974. Estudio sobre estados larvales de trematodes digeneos de peces Cyprinodontiformes. Physis, Sección B 33, 45–61.

Ostrowski de Núñez, M. 1976. Fauna de agua dulce en la República Argentina. IV. Las cercarias de *Ascocotyle* (*A.*) *tenuicollis* Price 1935 y de *Pygidiopsis pindoramensis* Travassos 1929 (Trematoda, Heterophyidae). Physis, Sección B 35, 51–57.

Ostrowski de Núñez, M. 1993. Life–history studies of heterophyid trematodes in the Neotropical Region: *Ascocotyle* (*Phagicola*) *diminuta* (Stunkard, Haviland, 1994) and *A.* (*P.*) *angrense* Travassos, 1916. *Syst. Parasitol.* 24, 191–199.

Ostrowski de Núñez, M. 2001. Life cycles of two new sibling species of *Ascocotyle* (*Ascocotyle*) (Digenea, Heterophyidae) in the Neotropical Region. Acta Parasitol*.* 46, 119–129.

Ostrowski de Núñez, M. 2017. Redescription of *Austrodiplostomum* *compactum* (Trematoda: Diplostomidae) from its type host and locality in Venezuela, and of *A. mordax* from Argentina. J. Parasitol. 103, 497–505.

Ostrowski de Núñez, M., Semenas, L., Brugni, N., Viozzi, G., Flores, V. 1999. Redescription of *Acanthostomoides apophalliformis* (Trematoda, Acanthostomidae) from *Percichthys trucha* (Pisces, Percichthyidae) with notes on its life cycle in Patagonia, Argentina. Acta Parasitol*.* 44, 222–228.

Ostrowski de Núñez, M., Brugni, N., Viozzi, G. 2000. *Polylekithum percai* n. sp. (Trematoda: Allocreadiidae) from *Percichthys trucha* (Perciformes: Percichthyidae) in Patagonia, Argentina, and a redescription of *Homalometron papilliferum* (Szidat, 1956) n. comb. Syst. Parasitol. 47, 51–57.

Ostrowski de Núñez, M., Flores, V., Viozzi, G., Kreiter, A. 2004. *Stephanoprora uruguayense* Holcman–Spector et Olagüe, 1989 (Digenea, Echinostomatidae) from Argentina, and comments on species of *Stephanoprora* from birds of the Neotropical region. Acta Parasitol*.* 49, 292–299.

Ostrowski de Núñez, M., Arredondo, N.J., Gil de Pertierra, A.A. 2017. Adult Trematodes (Platyhelminthes) of freshwater fishes from Argentina: a checklist. Rev.Suisse Zool. 124, 91–113.

Özer, A. 2002. Co–existence of *Dactylogyrus anchoratus* Dujardin, 1845 and *D. extensus* Mueller, Van Cleave, 1932 (Monogenea), parasites of common carp (*Cyprinus carpio*). Helmintologia 39, 45–50.

Paterson, R.A., Rauque, C.A., Fernández, M.V., Townsend, C.R., Poulin, R., Tompkins, D.M. 2013. Native fish avoid parasite spillback from multiple exotic hosts: consequences of host density and parasite competency. *Biol. Invasions* 15, 2205–2218.

Pearson, J. 2008. Heterophyidae. In: Bray, R.A., Gibson, D.I., Jones, A. (Eds.), Keys to the Trematoda, Vol. 3*.* CABI Publishing, Wallingford, pp. 113−141.

Popazoglo, F., Boeger, W.A. 2000. Neotropical Monogenoidea 37. Redescription of *Gyrodactylus superbus* (Szidat, 1973) comb. n. and description of two new species of *Gyrodactylus* (Gyrodactylidea: Gyrodactylidae) from *Corydoras paleatus* and *C. ehrhardti* (Teleostei: Siluriformes: Callichthyidae) of Southern Brazil. Folia Parasitol. 47, 105–110.

Quaggiotto, E.A., Valverde, F. 1992. Nuevas metacercarias del género *Tylodelphys* (Trematoda, Diplostomatidae) en poblaciones lacustres de *Galaxias maculatus* (Teleostei, Galaxiidae). Bol. Chil. Parasitol. 47, 19–24.

[Ramallo, G.](https://www.scopus.com/authid/detail.uri?authorId=6602414050&amp;eid=2-s2.0-0029171854), [Torres, P.](https://www.scopus.com/authid/detail.uri?authorId=7101784983&amp;eid=2-s2.0-0029171854) 1995. *Contracaecum* sp. larvae (Nematoda, Anisakidae) infection in *Salminus maxillosus* (Pisces, Characidae) in the dam of Termas de Río Hondo, Argentina. Bol. Chil. Parasitol. 50, 21–23.

Rauque, C., Semenas, L., Viozzi, G. 2002. Post–cyclic transmission in *Acanthocephalus tumescens* (Acanthocephala, Echinorhynchidae). Folia Parasitol*.* 49, 127–130.

Rauque, C., Viozzi, G., Semenas, L. 2003. Component population study of *Acanthocephalus tumescens* (Acanthocephala) in fishes from Lake Moreno (Argentina). Folia Parasitol*.* 50, 72–78.

Rauque, C., Semenas, L., Viozzi, G. 2006. Seasonality of recruitment and reproduction of *Acanthocephalus tumescens* (Acanthocephala) in fishes from Lake Moreno (Patagonia, Argentina). J. Parasitol. 92, 1265–1269.

Revenga, J.E. (1993) *Diphyllobothrium dendriticum* and *Diphyllobothrium latum* in fishes from Southern Argentina: association, abundance, distribution, pathological effects, and risk of human infection. J. Parasitol. 79, 379–383.

Revenga, J., Scheinert, P. 1999. Infections by helminth parasites in “Puyenes”, *Galaxias maculatus* (Galaxiidae, Salmoniformes), from Southern Argentina with special reference to *Tylodelphys barilochensis* (Digenea, Platyhelminthes). Mem. I. Oswaldo Cruz 94, 605–609.

Revenga, J.E., Semenas, L.G. 1991. Difilobotriasis en salmónidos introducidos en el Parque y Reserva Nacional Nahuel Huapi, Argentina: Morfología de plerocercoides. Arch. Med. Vet. 223, 157–164.

Revenga, J.E., Perfumo, C.J., Úbeda, C.A., Semenas, L.G. 1995. Difilobotriasis en salmónidos introducidos en el Parque y Reserva Nacional Nahuel Huapi, Argenina: patología de las lesiones producidas por *Diphyllobothrium* spp. Arch. Med. Vet. 27, 115–122.

Revenga, J.E., Torres, P.F., Baiz, M. 2005. Impact of a caged–trout farm on parasites of *Galaxias maculatus* in Lake Moreno, Southern Argentina. J. Parasitol. 91, 707–709.

Revenga, J.E., Torres, P.F., Siegmund, I. 2006a. *Acanthostomoides apophalliformis* (Trematoda: Cryptogonimidae) does not cause detectable mortality in *Galaxias maculatus* (Teleostomi: Galaxiidae). Braz. Arch. Biol. Techn. 49, 713–715.

Revenga, J.E., Torres, P.F., Siegmund, I. 2006b. *Galaxias maculatus* (Galaxiidae, Salmoniformes) infected with *Acanthostomoides apophalliformis* (Digenea, Platyhelminthes) in Southern Argentina. Pathology and absence of parasite induced mortality. Braz. J. Vet. Res. Anim. Sc. 43, 642–646.

Reyes–Piraino, X. 1982. Presencia de *Hepatoxylon trichiuri* (holten, 1802) (Cestoda: Trypano­rhyncha) en *Oncorhynchus tshawytscha* y *Somniosus pacificus* capturados en Chile. Investigaciones Marinas (Valparaíso) 10, 41–43.

Ritossa, L., Flores, V., Viozzi, G. 2013. Life–cycle stages of a *Posthodiplostomum* species (Digenea: Diplostomidae) from Patagonia, Argentina. J. Parasitol*.* 99, 777–780.

Ritossa, L., Flores, V., Viozzi, G. 2014. Infection dynamics of *Posthodiplostomum* sp. (Digenea: Diplostomidae) in first and second intermediate hosts from an Andean Patagonian lake (Argentina). Rev. Arg. Parasitol. 3, 16–23.

Rogers, W.A. 1968. *Pseudacolpenteron pavlovskyi* Bychowsky and Gussev, 1955 (Monogenea), from North America, with notes on its taxonomic status. J. Parasitol. 54, 339.

Rossin, M.A., Timi, J.T. 2014. *Characithecium* (Monogenoidea: Dactylogyridae) parasitic on the Neotropical fish *Oligosarcus jenynsii* (Teleostei: Characidae) from the Pampasic Region, Argentina, with the emendation of the Genus. Zootoxa 3893, 382–396.

Ruiz, A.E. 2007. Biología del pejerrey patagónico en el Embalse Florentino Ameghino, Chubut, Argentina. Universitas, Córdoba.

Salgado–Maldonado, G. 1980. Acantocéfalos de Aves I. Sobre la morfología de *Arhytmorhynchus brevis* Van Cleave, 1916 (Acanthocephala: Polymorphidae). An.Inst. Biol. Serie Zoología 51, 85–94.

Salgado–Maldonado G. 2006. Checklist of helminth parasites of freshwater fishes from Mexico. Zootaxa 1324, 1–357.

Scholz, T., Kuchta, R., Williams, C. 2012. *Bothriocephalus acheilognathi*. In: Woo, P.T.K., Buchmann, K. (Eds.), Fish Parasites: Pathobiology and Protection*.* CABI, Wallingford, pp. 282–297.

Semenas, L. 1998. Primer registro de diplostomiasis ocular en trucha arco iris cultivada en Patagonia (Argentina). Arch. Med. Vet. 30, 165–170.

Semenas, L. 2006. *Diphyllobothrium* spp. In: Basualdo, J.A., Coto, C.E., de Torres, R.A. (Eds.), Microbiología Biomédica. Editorial Atlante, Buenos Aires, pp. 1269–1274.

Semenas, L., Trejo, A. 1997. Redescription of *Acanthocephalus tumescens* (von Linstow, 1896) (Palaeacanthocephala: Echinorhynchidae) in *Galaxias maculatus* (Pisces: Galaxiidae) in Patagonia (Argentina). *Syst. Parasitol.* 36, 13–16.

Semenas, L., Ortubay, S., Úbeda, C. 1992. Studies on the development and life history of *Pomphorhynchus patagonicus* Ortubay, Úbeda, Semenas et Kennedy, 1991 (Palaeacanthocephala). Res.Rev. Parasitol. 52, 89–93.

Shimazu, T., Urawa, S., Coria, C.O. 2000. Four species of digeneans, including *Allocreadium patagonicum* sp. n. (Allocreadiidae), from freshwater fishes of Patagonia, Argentina. Folia Parasitol. 47, 111–117.

Suriano D.M. 1986. *Philocorydoras platensis* gen. n. et sp. n. (Monogenea: Ancryocephalidae) from *Corydoras paleatus* (Jenyns) (Pices: Callichthyidae) in Laguna Chascomus, República Argentina. Helmintologia 23, 249–256.

Szidat, L. 1956. Über die Parasitenfauna von *Percichthys trucha* (Cuv., Val.) Girard der patagonischen Gewässer und die Beziehungen den Wirtsfisches und seiner Parasiten zur Paläarktischen. Arch. Hydrobiol. 51, 542–577.

Szidat, L. 1964. Vergleichende helminthologische Untersuchungen an den Argentinischen Grossmöwen *Larus marinus dominicanus* Lichtenstein und *Larus ribidundus maculipennis* Lichtenstein nebst neven Beobachtungen über die Artbildung bei Parasiten. Z. Parasitenk. 24, 351–414.

Szidat, L. 1973. Morphologie und Verhalten von *Paragyrodactylus superbus* n.g., n.sp. Erregener eines Fischsterbens in Argentinien. Angew. Parasitol. 14, 1–10.

Szidat, L., Nani, A. 1951. Diplostomiasis cerebralis del pejerrey. Una grave epizootia que afecta a la economía nacional producida por larvas de trematodes que destruyen el cerebro de los pejerreyes. Rev.Mus.Arg. Cs. Nat. 1, 324–383.

Szidat, L., Nani, A. 1952. Nota preliminar sobre una parasitosis grave de los pejerreyes producida por larvas de trematodes de la familia Diplostomidae Poirier 1886 que destruyen el cerebro de los peces. Rev.Med. Vet. 34, 9–14.

Szidat, L., Soria, M.F. 1952. Difilobotriasis en nuestro país. Nota preliminar. La Prensa Médica Argentina, 39, 77–78.

Szidat, L., Soria, M.F. 1957. Difilobotriasis en nuestro país. Sobre una nueva especie de *Sparganum*, parasita de salmones, y de *Diphyllobothrium*, parasita de gaviotas, del lago Nahuel Huapi. Bol. Mus. Arg. Cs. Nat. Bernardino Rivadavia 9, 1–22.

Tanzola, R.D., Guagliardo, S., Romero, A., Schwerdt, C., Schwerdt, M., Galeano, N. 2009. Diversidad parasitaria en peces de agua dulce del sudoeste de la provincia de Buenos Aires. In: Cazzaniga, N., Arelovich, H.M. (Eds.), Ambientes y recursos naturales del sudoeste bonaerense: Producción, contaminación y conservación*.* Actas de las V Jornadas Interdisciplinarias del Sudoeste Bonaerense, Editorial Ediuns, Bahía Blanca, pp. 381–394.

Torres, P. 2013. Difilobotriasis. In: Apt, W. (Ed.), Parasitología humana. Mc Graw Hill Education, México, pp. 202–213.

Torres, P., Teuber, S., Miranda, J.C. 1990. Parasitismo en ecosistemas de agua dulce de Chile. 2. Nematodos parásitos de *Percichthys trucha* (Pisces: Serranidae) con la descripción de una nueva especie de *Camallanus* (Nematoda: Spiruroidea). Stud.Neotr. Fauna Env. 25, 111–119.

Torres, P., Contreras, A., Cubillos, V., Gesche, W., Montefusco, A., Rebolledo, C., Mira, A., Arenas, J., Miranda, J.C., Asenjo, S., Schlatter, R. 1992. Parasitismo en peces, aves piscívoras y comunidades humanas ribereñas de los lagos Yelcho y Tagua–Tagua, X Región de Chile. Arch. Med. Vet. 24, 77–92.

Trejo, A. 1992. A comparative study of the host–parasite relationship of *Pomphorhynchus patagonicus* (Acanthocephala) in two species of fish from Lake Rosario (Chubut, Argentina). *J. Parasitol.* 78, 711–715.

Trejo, A. 1994. Observations on the host specificity of *Pomphorhynchus patagonicus* (Acanthocephala) from Alicura Reservoir (Patagonia, Argentina). J. Parasitol. 80, 829–830.

Trejo, A., Semenas, L., Viozzi, G. 2000. *Acanthocephalus tumescens* (Acanthocephala, Echinorhynchidae) in *Galaxias maculatus* (Pisces, Galaxiidae) of lake Gutiérrez, Patagonia, Argentina. *J. Parasitol.* 86: 188–191.

Úbeda, C., Trejo, A., Semenas, L., Ortubay, S. 1994. Status of three different fish hosts of *Pomphorhynchus patagonicus* Ortubay, Úbeda, Semenas et Kennedy, 1991 (Acanthocephala) in Lake Rosario (Argentina). Res.Rev. Parasitol. 54, 87–92.

Velázquez-Velázquez, E., González-Solís, D., Salgado-Maldonado, G. 2011. *Bothriocephalus acheilognathi* (Cestoda) in the endangered fish *Profundulus hildebrandi* (Cyprinodontiformes), Mexico. Rev. Biol. Trop. 59, 1099–1104.

Viozzi, G., Brugni, N. 2004. *Duplaccessorius andinus* n. gen., n. sp. (Dactylogyridae: Ancyrocephalinae) from the gills of *Percichthys trucha* (Perciformes: Percichthyidae) in Patagonia (Argentina). J. Parasitol. 90, 966–969.

Viozzi, G., Flores, V. 2002. Population dynamics of *Tylodelphys* *destructor* and *Diplostomum* (*Austrodiplostomum*) *mordax* (Digenea: Diplostomidae) co–ocurring in the brain of *Odontesthes* *hatcheri* (Osteichthyes: Atherinidae) from Lake Pellegrini, Patagonia, Argentina. J.Wildlife Dis. 38, 4, 784–789.

Viozzi, G., Flores, V., Ostrowski de Núñez, M. 2000. *Steganoderma szidati* n. sp. (Trematoda: Zoogonidae) from *Galaxias maculatus* (Jenyns) and *G. platei* Steindachner in Patagonia, Argentina. Syst. Parasitol. 46, 203–208.

Viozzi, G., Semenas, L., Brugni, N., Flores, V. 2009. Metazoan parasites of *Galaxias maculatus* (Osmeriformes: Galaxiidae) from Argentinean Patagonia. Comp. Parasitol. 76, 229–239.

von Linstow, V. 1896. Nemathelminthen. Hamburger Magalhaensische Sammelreise, 1–22.

Waicheim, A., Rauque, C.A., Viozzi, G.P., Blasetti, G., Cordero, P. 2014. Macroparasites of the invasive fish, *Cyprinus carpio*, in Patagonia, Argentina*. Comp. Parasitol.* 81, 270–275.

Yamaguti, S. 1963. Systema Helminthum: Monogenea and Aspidocotylea. Vol. IV. Interscience Publishers, New York.

Yamaguti, S. 1971. Systema Helminthum: Sinopsis of Digenetic Trematodes of Vertebrales. Vol. 1. Keigaku Publishing, Tokio.
